# Structural and Drug Screening Analysis of the Non-structural Proteins of Severe Acute Respiratory Syndrome Coronavirus 2 Virus Extracted From Indian Coronavirus Disease 2019 Patients

**DOI:** 10.3389/fgene.2021.626642

**Published:** 2021-03-09

**Authors:** Nupur Biswas, Krishna Kumar, Priyanka Mallick, Subhrangshu Das, Izaz Monir Kamal, Sarpita Bose, Anindita Choudhury, Saikat Chakrabarti

**Affiliations:** Structural Biology and Bioinformatics Division, Council for Scientific and Industrial Research (CSIR)–Indian Institute of Chemical Biology (IICB), Kolkata, India

**Keywords:** SARS-CoV2, COVID-19, non-structural proteins, database, mutation

## Abstract

The novel coronavirus 2 (nCoV2) outbreaks took place in December 2019 in Wuhan City, Hubei Province, China. It continued to spread worldwide in an unprecedented manner, bringing the whole world to a lockdown and causing severe loss of life and economic stability. The coronavirus disease 2019 (COVID-19) pandemic has also affected India, infecting more than 10 million till 31st December 2020 and resulting in more than a hundred thousand deaths. In the absence of an effective vaccine, it is imperative to understand the phenotypic outcome of the genetic variants and subsequently the mode of action of its proteins with respect to human proteins and other bio-molecules. Availability of a large number of genomic and mutational data extracted from the nCoV2 virus infecting Indian patients in a public repository provided an opportunity to understand and analyze the specific variations of the virus in India and their impact in broader perspectives. Non-structural proteins (NSPs) of severe acute respiratory syndrome coronavirus 2 (SARS-CoV2) virus play a major role in its survival as well as virulence power. Here, we provide a detailed overview of the SARS-CoV2 NSPs including primary and secondary structural information, mutational frequency of the Indian and Wuhan variants, phylogenetic profiles, three-dimensional (3D) structural perspectives using homology modeling and molecular dynamics analyses for wild-type and selected variants, host-interactome analysis and viral–host protein complexes, and *in silico* drug screening with known antivirals and other drugs against the SARS-CoV2 NSPs isolated from the variants found within Indian patients across various regions of the country. All this information is categorized in the form of a database named, Database of NSPs of India specific Novel Coronavirus (DbNSP InC), which is freely available at http://www.hpppi.iicb.res.in/covid19/index.php.

## Introduction

Severe acute respiratory syndrome coronavirus 2 (SARS-CoV2) is responsible for the global pandemic of coronavirus disease 2019 (COVID-19) (Gorbalenya et al., 2020). The SARS-CoV2 is an enveloped non-segmented positive sense single-stranded RNA virus. It belongs to the Nidovirales order and Coronaviridae family (Fehr and Perlman, 2015). Its genomic length is ∼29,900 base pairs, making it one of the largest known RNA virus genomes (Fehr and Perlman, 2015; NC_045512, 2020). The genomic structure contains a 5′ cap structure and 3′ ploy(A) tail with 11 open reading frames (ORFs). One major characteristic feature of SARS-CoV2 genome is that almost two-thirds of the genome (∼20 kb) corresponds to the replicase gene (ORF1ab), which expresses a polyprotein. The remaining part of the genome ∼10 kb encodes other structural and accessory proteins. The replicase gene is followed by the ORF2 spike glycoprotein (S), ORF3a, ORF4 envelope (E) gene, ORF5 membrane (M) gene, ORF6, ORF7a, ORF7b, ORF8, ORF9 nucleocapsid phosphoprotein (N), and ORF10 (Wu et al., 2020; Yoshimoto, 2020). Among these, spike, envelope, membrane, and nucleocapsid proteins are the structural proteins, while the rest are accessory proteins. The ORF1ab polyprotein is composed of 16 non-structural proteins (NSPs).

The NSPs of any virus are encoded by the virus genome but are not included in the virus particle. For coronaviruses, NSPs play important roles in RNA synthesis and processing, helping in its survival as well as virulence power (Snijder et al., 2016). For SARS-CoV2, the first NSP (NSP1), also known as the leader protein, binds with 40S ribosomal subunit and plays an inhibitory role in mRNA translation (Narayanan et al., 2020; Thoms et al., 2020). The second NSP, NSP2, binds with host proteins and disrupts host cell environment (Angeletti et al., 2020; Yoshimoto, 2020). The third NSP (NSP3), the longest protein of SARS-CoV2, has 1,945 amino acids and is a papain-like protease. NSP3 plays multiple roles in host cells, including regulation of IRF3 and NF-kappaB signaling (Frieman et al., 2009). NSP3, NSP4, and NSP6 together play a role in host membrane rearrangements necessary for viral replication (Angelini et al., 2013). NSP5 is a 3C-like protease and cleaves at 11 distinct sites of the polyprotein to yield other NSPs (Muramatsu et al., 2016; Yoshimoto, 2020). NSP6 is known to locate at endoplasmic reticulum and generates autophagosomes (Forni et al., 2017; Benvenuto et al., 2020). The NSP7–NSP8 cofactors and NSP12 catalytic subunits create the core polymerase complex (Peng et al., 2020; Wang et al., 2020). Apart from creating complex with NSP7, NSP8 creates complex with accessory protein ORF6 also (Kumar et al., 2007). Both NSP9 and NSP10 are small non-enzymatic proteins and assist in the function of NSP12 (Zhang et al., 2020). NSP10 also interacts with NSP14 and NSP16. The NSP16–NSP10 complex provides protection to the virus from the host’s innate immune system (Lin et al., 2020; Viswanathan et al., 2020). NSP11 consists of only 13 amino acids, of which the first nine are identical to the first nine amino acids of NSP12 (Yoshimoto, 2020). NSP12 is the RNA-directed RNA polymerase (RdRp) and is responsible for the replication and transcription of the RNA genome. Several probable drugs, including remdesivir, are targeted to NSP12 (Shannon et al., 2020). NSP13 is the helicase protein, and its binding with NSP12 enhances helicase activity (Yoshimoto, 2020). NSP13, NSP14, and NSP15 can suppress interferon production and host signaling (Yuen et al., 2020). NSP14 is the guanine-N7 methyltransferase and plays a vital role in the RNA replication process (Romano et al., 2020). NSP15 is the endoribonuclease and is also a probable target of various drugs. NSP16 is the 2’-*O*-methyltransferase. Both NSP14 and NSP16 play vital roles in creating RNA cap in the viral genome (Krafcikova et al., 2020). Due to their pivotal roles in the replication as well as in the life cycle of SARS-CoV2, it is important to study the frequency, nature, and probable outcomes of the mutations that are being observed at the NSP regions of the virus.

The COVID-19 pandemic has spread in India, the second most populated country in the world. The total number of infected persons is 10,266,674 on 31 December 2020, which resulted in 148,738 deaths (Ministry of Health and Family Welfare Goverment of India, 2020) along with enormous socioeconomic disturbance (Gopalan and Misra, 2020), and the situation remains alarming to date. In this context, we have focused on the sequences of NSPs of SARS-CoV2 extracted from Indian patients and created a database, Database of NSPs of India specific Novel Coronavirus (DbNSP InC). In this manuscript, we are reporting our database, DbNSP InC, which provides exhaustive information on the NSPs of SARS-CoV2 observed in Indian patients. It provides the functional information; mutations observed in Indian patients samples; comparison of mutations with the Wuhan samples; primary and secondary structural analyses; strain and mutation analyses; and mutations observed in the deceased, mild, and asymptomatic patients samples along with the distribution of mutations across different Indian states and phylogenetic analysis. DbNSP InC is enriched with three-dimensional (3D)/tertiary structures of wild-type (WT) and mutated NSPs. The information on host protein interaction is also provided as interactive interactome networks of NSPs with host proteins and structure of host protein complexes. Molecular dynamics (MD) analysis was also performed in order to investigate the stability of the proposed complexes. *In silico* drug screening with known antiviral and other drugs was performed against the SARS-CoV2 NSPs isolated from the variants found within Indian patients across various regions of the country. The database is freely available at http://www.hpppi.iicb.res.in/covid19/index.php.

## Materials and Methods

### Sequence and Mutation Data Collection

The protein sequences of SARS-CoV2 virus were collected from the EpiCoV database of GISAID (2020). The database was searched up to 8 October 2020 using keywords “hCoV-19”, “India”, and “human”. It provided 2,338 complete and high-coverage nucleotide sequences. Sequences with genomes > 29,000 bp were considered complete. Sequences with <1% Ns (undefined bases) were considered as high-coverage sequences. Corresponding protein sequences for different NSPs were extracted. Database specific renaming (code) was done for each sequence based on the Indian state from where it was collected. Additional metadata for the sequences, which include location of sample collection, patient status, and other relevant information, were also collected.

Along with the sequences from India, human coronavirus 2019 (hCoV-19) sequences for samples collected from Wuhan, China, from where the pandemic initiated were also extracted from the GISAID database. Search with keywords “hCoV-19”, “China/Wuhan/”, and “human” yielded 255 sequences, which were used in our analysis. Sequences from different continents (North America, South America, Europe, Africa, Asia, and Oceania) were also collected in a similar fashion from the GISAID database, for comparing frequencies of the most frequent mutations of Indian samples in the global context. National Center for Biotechnology Information (NCBI) reference sequence NC_405512.2 (NC_045512, 2020) was considered as a reference sequence for calling the mutations. These sequences (NC_405512.2) were collected from the human sample in Wuhan, China, in December 2019.

### Alignments, Phylogeny, and Mutation Frequency Calculation

Redundancy filter criteria via CD-HIT server (Fu et al., 2012) were applied to extract unique representative NSP sequences and to exclude redundant sequences, for each NSP of protein family. The number of CD-HIT runs was kept one, with sequence identity cutoff 1.0 (100% identity). It provided clusters of sequences that are less than 100% identical. The cluster representative sequences along with the NCBI reference sequence were aligned using the MUSCLE protein sequence alignment tool (Madeira et al., 2019). MUSCLE also constructed a phylogenetic tree for the cluster representative sequences. The tree files in the *newick* format were further used to construct an interactive phylogenetic tree using javascripts file phylotree.js (Shank et al., 2018). In-house python (version 3.4) codes were used for extracting mutations from alignment data files and calculating mutation frequencies.

### Metadata Analysis

Using the metadata of disease severity status of patients, we analyzed the association of different mutations with disease severity status. Fisher’s exact test was performed using the following contingency table (Hoffman, 2019) for deceased samples,


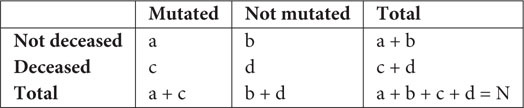


where *N* is the total number of sequences. Similar tables were used for mild and asymptomatic samples. The probability of obtaining a given set of result, *p*-value, is provided by a hypergeometric distribution,

(1) $$p=\frac{\left(\begin{array}{c} a+c\\ a \end{array}\right)\,\left(\begin{array}{c} b+d\\ b \end{array}\right)}{\left(\begin{array}{c} N\\ a+b \end{array}\right)}$$

where $\left(\begin{array}{c} i\\ j \end{array}\right)$ denotes binomial coefficient of any given variable *i* and *j*.

### Strain Specific Mutational Count and Substitution Score Calculation

Distributions of mutation frequencies for Indian sequences were estimated according to their prevalence in various Indian states as the origin of the infected patients. The substitution scores for each cluster representative sequence were calculated using the point accepted mutation (PAM) matrix 250 (Dayhoff, 1969). The substitution scores are displayed as “*Strain and mutation analyses*” column in the DbNSP InC database. The cells are colored according to the substitution score of the observed mutations. Blank cell means no mutation was observed. All interactive plots were constructed using Google Chart API.

### Primary Structure Analysis and Secondary Structure Prediction

Primary structure analysis was done using the ProtParam tool of ExPASy server (Artimo et al., 2012) where information regarding amino acid sequence, molecular weight, isoelectric point (pI), amino acid composition, number of negatively and positively charged residues, instability index, aliphatic index, and average of hydropathicity of each reference NSP sequence are provided. Additionally, an option is implemented within the module where same information for NSP variants extracted from Indian patients can be retrieved via live search.

Similarly, secondary structure analysis was done using the PSIPRED program (Buchan and Jones, 2019) where the likelihood of each residue forming a helix, strand, or coil is provided along with a confidence score. For each protein, brief functional information, collected from the UniProt (The UniProt Consortium, 2019) was also provided.

### Structure Prediction of Wild-Type and Mutant Non-structural Proteins

SARS-CoV2 WT proteins for which the 3D structures are available were extracted from the Protein Data Bank (PDB) (Burley et al., 2019). 3D structures of WT NSPs for which structures are not available were modeled via homology modeling approach using the MODELER program (Webb and Sali, 2016). WT NSPs models were also collected from the Zhang lab COVID-19 resource (Zhang Lab, 2020) for comparison purposes.

Similarly, 3D models of the mutant (India specific) NSPs were generated using the MODELER. One hundred ensemble model structures were generated for each WT and mutant protein, and the best possible model was selected based on the MODELER DOPE score. All the 3D models were evaluated using various structure validation tools such as PROCHECK (Laskowski et al., 1993), ERRAT (Colovos and Yeates, 1993), Verify3D (Eisenberg et al., 1997), QMEAN (Benkert et al., 2011), and ProSA (Wiederstein and Sippl, 2007). Images of the protein structures were created by the CHIMERA software (Pettersen et al., 2004).

### Host Protein Interactome Network Analysis

The SARS-CoV2 NSP and human protein–protein interactome (PPI) network (PPIN) was constructed using the interaction data made available by Gordon et al. (2020a, b) and Biogrid (Stark et al., 2006). We have considered only experimentally validated interactions. A total of 802 human interactor proteins were extracted for 15 SARS-CoV2 NSPs. Further, first layer interactors of the human proteins were collected from the STRING (Szklarczyk et al., 2019) database (version 11).

With the use each of this network, a network analysis approach was implemented to identify five types of topologically important nodes (TINs), namely, hubs, central nodes (CNs), bottlenecks (BNs) (Yu et al., 2007), global network perturbing proteins (GNPPs), and local network perturbing proteins (LNPPs) (Bhattacharyya and Chakrabarti, 2015). Network and node indices like degree, betweenness, closeness, and clustering coefficients were calculated from the extracted viral–human PPIN for identifying the TINs. TINs were calculated using previously reported methods and protocols (Bhattacharyya and Chakrabarti, 2015).

A network representation of important nodes of these NSPs and human proteins network is displayed in an interactive 3D network viewer at the DbNSP InC database. Additional functional details about the important network proteins are made available via GeneCards (Stelzer et al., 2016) link embedded within the interaction viewer window. The network is constructed using javascript-based open source technologies (three.js and 3d-force-graph.js).

### Generation of Viral–Host Protein–Protein Interaction Complex

Three-dimensional structures (models) of the selected complexes of SARS-CoV2 NSPs and human proteins (with known 3D structures) were predicted by a widely used protein docking program, PatchDock (Schneidman-Duhovny et al., 2005). PatchDock allows geometric shape complementarity matching with the help of geometric hashing and pose-clustering techniques. The top 100 solutions from PatchDock-based docking score were clustered according to the root mean square deviation (RMSD) in CHIMERA software (Pettersen et al., 2004) to determine the largest docked clusters. The top scoring solution from the largest cluster was selected as representative pose with the assumption that clusters having a higher number of similar frames are more likely to possess the best possible interaction pose.

One hundred and thirteen complex structures were generated using seven known NSP structures and 41 predicted (5 WT and 36 mutant) NSP proteins with 28 human proteins of known structures. The human proteins were chosen based on the availability of high-quality crystal structures.

PISA software (Krissinel and Henrick, 2007) was used to calculate the structural and chemical properties of the macromolecular interfaces such as interface area, free energy of dissociation, presence of hydrogen bond and salt bridges. The strength of the binding at the interface was estimated via free energy of formation (ΔG_*int*_) and solvation energy (SE) gain (ΔG_*solv*_). Various types of molecular interactions, such as hydrogen bond and salt bridges, formed by the two interacting chains at the interface were also calculated and provided within the respective window of the complexes at the DbNSP InC database.

Calculation of fraction of conserved native contacts (FNATs) with respect to a reference complex/interface is a standard complex evaluation criterion. FNAT is the number of native (correct) residue–residue contacts in the docked (predicted) complex divided by the number of contacts in the original (known). According to Critical Assessment of PRedicted Interactions (CAPRI) (Lensink et al., 2020) criteria, predicted complexes with 10% ≤ FNAT <30% are regarded as acceptable predictions, 30% ≤ FNAT <50% as medium-quality predictions, and FNAT ≥50% as high-quality predictions. In this case, we have evaluated the alteration of the interface formed by the mutant NSPs with respect to the WT protein complex via calculation of FNAT. FNAT values of both the chains forming the complex are provided in the DbNSP InC database.

### Molecular Dynamics Analysis

The 3D structures of WT and mutant NSPs as well as complexes of NSPs (WT and mutant) and human proteins were subjected to MD simulation to study the impact of mutation on the structural dynamics by using the Desmond (Bowers et al., 2006) MD simulation package. Further, MD simulations of the NSPs complexed (docked) with antiviral drugs were also performed using the GROMACSv4.5.3 simulation package (Abraham et al., 2015) to understand the structural and energetic stabilities of the proposed protein–drug complexes.

In Desmond (Krissinel and Henrick, 2007) MD simulations, *OPLS_2005* force field parameters (Kaminski et al., 2001) were used to generate the coordinates and topology of the molecules. The system was solvated with TIP3P (Mark and Nilsson, 2001) water, and counter ions were added to neutralize the overall charge of the system. Orthorhombic periodic boundary conditions were defined to specify the shape and size of the simulation box buffered at 10-Å distances from the molecules. A hybrid method combining the steepest decent and the limited-memory Broyden–Fletcher–Goldfarb–Shanno (LBFGS) algorithm (Saputro and Widyaningsih, 2017) was used to minimize the energy of the system. Further, the system was equilibrated in NVT followed by NPT conditions using default protocol of Desmond. Finally, the production run was performed at 300K temperature and 1 atm pressure with a time step of 2 fs for 200 ns. The temperature and pressure of NPT ensemble were regulated by using Nosé–Hoover chain thermostat (Evans and Holian, 1985) and Martyna–Tobias–Klein barostat (Martyna et al., 1994), respectively. Reversible reference system propagator algorithms (RESPA) (Tuckerman et al., 1992) was used for integrating the equations of motion. Trajectories were recorded at every 4.8 ps and analyzed by Desmond “simulation analysis tool.” Energy profile during simulation was analyzed by “simulation quality analysis tool” of Desmond package. RMSD and root mean square fluctuations (RMSFs) of the protein residues were analyzed using the “simulation event analysis” module.

Each antiviral drug complexed with SARS-CoV2 NSPs obtained from docking analyses was subjected to MD simulation using the GROMACSv4.5.3 simulation package (Abraham et al., 2015). Coordinates and topology files of receptor molecule were generated with *Amberff99sb* force field (Case et al., 2005). The topology and coordinate files of ligands were generated using ACPYPE (AnteChamber PYthon Parser interface) (Sousa Da Silva and Vranken, 2012). A cubic simulation box was defined and filled with TIP3P water (Mark and Nilsson, 2001) molecules. Two-stage minimization of the system was performed using the steepest-descent (Nocedal and Wright, 2006) and conjugate-gradient (Straeter, 1971) minimization algorithms. The system was equilibrated under NVT (constant number of particles, volume, and temperature) and NPT (constant number of particles, pressure, and temperature) conditions for 500 ps at a temperature of 300K and 1 atm pressure. After equilibration step, final production run was performed under NPT condition for 10 ns at 300K temperature and 1 atm pressure. Trajectories were saved at the interval of 0.02 ps, and a total of 500,000 snapshots were recorded. A total of 100 snapshots, recorded at the interval of 100 ps, were used to calculate the binding free energy using *g_mmpbsa* tool (Kumari et al., 2014).

### High-Throughput Virtual Screening of Antivirals and Known Drugs Against the Novel Coronavirus 2 Non-structural Proteins

A high-throughput virtual screening (HTVS) technique was employed to identify the efficient binders of NSP structures that may serve as potential inhibitors for various NSPs. In this work, two different small molecule datasets were utilized to identify the potential binders. For the screening of first dataset, all known antiviral drugs (111 compounds) were collected from DrugBank (2020) database, were docked onto the NSP structures (NSP5, NSP12, NSP13, NSP14, NSP15, and NSP16), and were ranked by using all the fitness scores (GoldScore, ChemPLP, Chemscore, and ASP) of GOLD docking software (Jones et al., 1997). The GOLD software optimizes the fitness score of many possible docking solutions using a genetic algorithm. The following parameters were used in the docking cycles: population size (100), selection pressure (1.10), number of operations (100,000), number of islands (5), niche size (2), crossover weight (95), mutation weight (95), and migration weight (10). The docking scores were normalized to 0 to 1 scale by using the following formula:

(2) $$S\,c\,o\,r\,e_{N\,o\,r\,m\,a\,l\,i\,z\,e\,d}=\frac{\left(S-S_{m\,i\,n}\right)}{\left(S_{m\,a\,x}-S_{\min}\right)}$$

where *S* is raw docking score of a particular molecule, and *S*_*max*_ and *S*_*min*_ are the maximum and minimum docking scores in the top quartile solutions, respectively.

For the screening of second dataset, all the small molecule known drugs and/or drug-like substances available in the DrugBank (2020) database (8,736 compounds) were extracted, and the same strategy used for the screening of antiviral drugs (described above) was followed to identify the potential inhibitors for NSP structures.

Antivirals and known drug molecules commonly appearing (at least in three scoring schemes) among the top 25% solutions of each fitness score were considered as probable inhibitors of the target SARS-CoV2 NSPs. The probable inhibitors were identified and ranked based on the average normalized score. All the probable inhibitors identified from the antiviral drug dataset were subjected to MD simulation followed by binding free energy calculation to check the stability of the protein–ligand complex.

## Results

### Mutational Frequency Analysis of the Indian and Wuhan Novel Coronavirus 2 Variants

**FIGURE 1:**
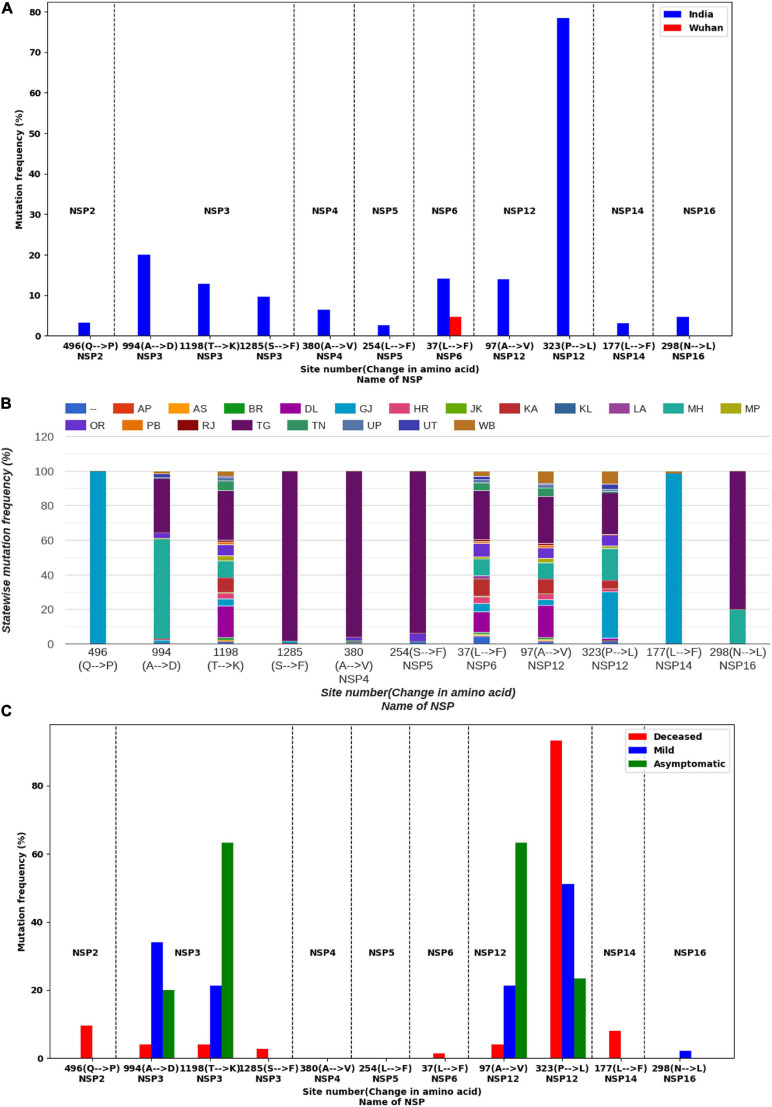
Mutation analysis of non-structural proteins (NSPs) of severe acute respiratory syndrome coronavirus 2 (SARS-CoV2) sequences having frequencies ≥2.5% in India. **(A)** Comparison of mutation frequencies for different NSPs from samples collected from India (blue) and Wuhan, China (red). Dashed lines are drawn to separate NSPs. **(B)** Distribution of mutations in different Indian states. **(C)** Occurrence of mutations in different types of patients.

Mutations were identified within the sequences of NSPs collected from India and Wuhan, China. The mutation frequencies were calculated, and their distribution plots for each NSP are displayed in the database DbNSP InC under the column “*Mutation frequency*.” Higher (≥2.5% of the total 2,338 samples) frequencies of mutations in NSPs from the Indian samples were observed especially for NSP2, NSP3, NSP4, NSP5, NSP6, NSP12, NSP14, and NSP16. On the other hand, NSP1, NSP7, NSP8, NSP9, NSP10, NSP13, and NSP15 show lower mutation frequencies (<2.5%) for the Indian samples. Figure 1A lists the mutations for different NSPs within the Indian population where the mutation frequency is more than 2.5%.

We observed in NSP12 that the RdRp has the most observed mutations at site 323, having a mutation frequency of 78.44% and that the mutation is from amino acid proline (P) to leucine (L). NSP12 sequences possess another mutation at site 97(A→V) having a frequency of 13.9%. NSP3 is the longest NSP and has a maximum number of mutations. The highest mutation frequency (20.02%) observed for NSP3 is at 994(A→D). NSP3 has two more frequently mutated sites, 1198(T→K) having a mutation frequency of 12.75% and 1285(S→F) 9.58% frequency. NSP2 has a mutation at site 496(Q→P) of 3.21% frequency. NSP4 has a mutation at site 380(A→V) with a frequency of 6.42%, while NSP5 has a mutation at site 254(S→F) with a frequency of 2.65%. Similarly, NSP6, NSP14, and NSP16 have mutations at the sites 37(L→F), 177(L→F), and 298(N→L) with mutation frequencies of 14.16, 3.12, and 4.66%, respectively.

We compared the mutations observed in Indian sequences with the mutations observed in Wuhan sequences and found significant differences in these two types of samples (Figure 1A). For NSP1, mutation frequencies are low for both the Indian and Wuhan samples. However, for NSP2, site 198(V→I) has been mutated in 2.75% of the Wuhan samples and 1.37% of the Indian samples (Supplementary Figure S1). No mutation was observed at site 496 of NSP2 for the Wuhan samples, indicating that a mutation at 496(Q→P) is specific to the Indian samples. For NSP3, the Wuhan samples showed a mutation at 1937(T→I), which was not observed in the Indian samples (Supplementary Figure S1). However, the Indian samples have shown three highly mutated sites (994, 1198, and 1285) as shown in Figure 1A. On the other hand, 230(E→G) site of NSP4 has 3.53% mutation frequency for the Wuhan samples and no mutation for the Indian samples (Supplementary Figure S1). Similarly, site 120(G→C) of NSP5 has 3.14% mutations for the Wuhan samples but no mutations for the Indian samples. For NSP6, a mutation at 37(L→F) was observed for both the Indian and Wuhan samples, having frequencies of 14.16 and 4.71%, respectively. NSP7, NSP8, NSP9, and NSP10 appear to have very low mutating sites for both the Indian and Wuhan samples. For the Wuhan samples, NSP12 mutated only at site 415(F→S) with a frequency of 6.67% (Supplementary Figure S1). NSP13, NSP14, NSP15, and NSP16 showed a mutation frequency <2.5% for the Wuhan samples.

### State-Wise and Strain-Wise Mutational Analyses of the Indian Variants

We analyzed the presence of mutations across samples collected from different Indian states. The information of the state was not available for some samples, which are marked as “–” in the DbNSP InC database. Other state names are mentioned in an abbreviated form. The abbreviation information is provided at the “Info” page of the database.

**FIGURE 2:**
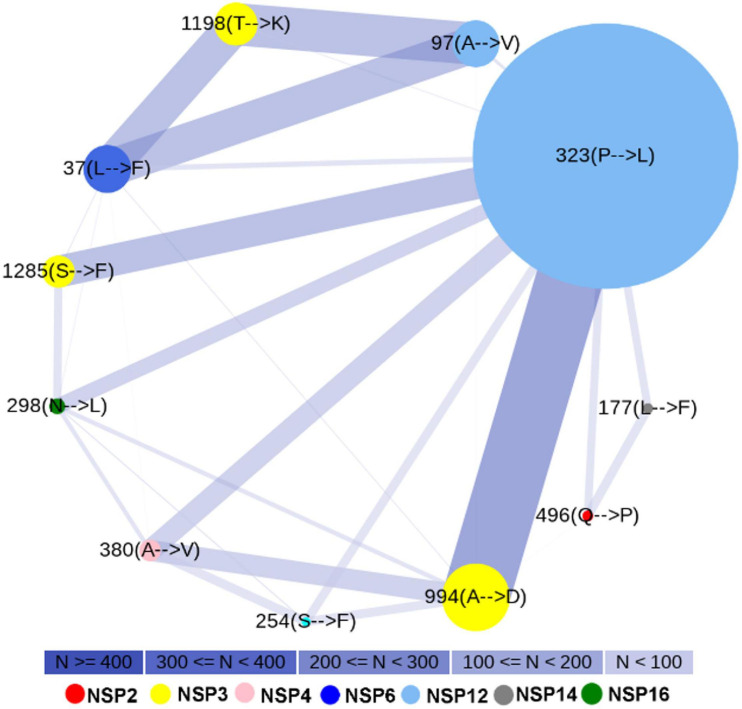
Co-occurrence of most frequent mutations. Node color indicates different non-structural proteins (NSPs). Node size indicates mutation frequency. The smallest node corresponds to 62 sequences, while the largest node corresponds to 1,834 sequences. Edge width and color represent the number of sequences (N) where the mutations have co-occurred.

We observed marked differences in the mutation frequency across the Indian states, indicating regional accumulation of certain mutation types. Figure 1B shows the state-wise appearances of different mutations. Figure 2 shows the co-occurrence of mutations across different samples. For example, two major mutating sites, 994(A→D) and 1198(T→K), for NSP3 never co-appeared in the same sample. We also noticed that 57.69% of mutations at 994(A→D) was observed in Maharashtra (MH) state (Figure 1B). For mutation 1198(T→K), 28.52% mutations appeared at samples from the state of Telangana (TG) and 18.46% from Delhi (DL). Similar accumulation of certain mutation types was noticed in NSP12 also. The most frequent variant within Indian patients [NSP12: 323(P→L)] has 26.72% representation from the state of Gujarat (GJ), followed by TG (24.21%) and MH (18.21%) (Figure 1B). However, for site 97, only 3.38% mutations were observed at samples from GJ and 9.23% for MH. TG has the highest contribution (27.08%) for a mutation at site 97. It indicates that sequences having a mutation at 323 have a tendency of not to be mutated at site 97. However, West Bengal (WB) shares 7.38 and 7.58% of mutations at sites 97 and 323, respectively, indicating a possible co-occurrence of these two mutations. The strain-wise analysis also revealed similar features of the mutual exclusiveness of mutations at sites 97 and 323 for sequences from GJ and TG. We observed 22 sequences have a mutation at both sites 97 and 323. Out of these 22 sequences, 15 are from WB indicated the existence of a variant of NSP12 where both 97 and 323 sites are mutated.

Figure 2 shows the existence of a broad edge between 994(A→D) of NSP3 and 323(P→L) of NSP12, which is due to their co-occurrence in 19.76% samples. We observed that mutations 1198(T→K) of NSP3 and 97(A→V) of NSP12 occurred simultaneously at 12.49% of samples. Two other broad edges are connected with 37(L→F) of NSP6. These are due to a co-occurrence of 37(L→F) of NSP6 with 1198(T→K) of NSP3 in 10.91% samples and a co-occurrence of 37(L→F) of NSP6 with 97(A→V) of NSP12 in 10.95% samples.

From the PAM 250 matrix (Dayhoff, 1969), we observed that the substitution scores for T→K, A→V, and A→D are 0, indicating that the mutations are tolerable whereas the substitution score of -3 at 323(P→L) mutation (Supplementary Figure S2) indicates probable deleterious impact. We observed mutations 323(P→L) of NSP12 and 1285(S→F) of NSP3, both having substitution scores of -3, which co-occurred at 4.94% samples (Supplementary Figure S2 and Figure 2). Mutations 323(P→L) of NSP12 and 298(N→L) of NSP16, both having substitution scores of -3, co-occurred at 4.53% samples. Mutations 496(Q→P) of NSP2 and 380(A→V) of NSP4 have substitution scores of 0. On the other hand, L→F mutation observed at site 37 of NSP6 and at 177 of NSP14 has a substitution score of +2 (Supplementary Figure S2).

### Patient Status and Disease Severity-Wise Mutational Analysis

We further analyzed the metadata available with the sequencing data in order to associate the observed mutations with the clinical status/manifestation of the patients. We found, out of 2,338 sequences, that the patient status of 74 sequences was marked as deceased. Forty-seven sequences had patient status “mild,” and 30 were marked as “asymptomatic.” We analyzed the mutations in these samples, and comparative plots of occurrence of mutations for these three types of samples are provided in the DbNSP InC database as “*Mutation in different types of patients*” for different NSPs and are partially reconstructed in Figure 1C. We observed that NSP2 mutation 496(Q→P) was present in 9.46% of deceased samples. For NSP3, both mutations 994(A→D) and 1198(T→K) are mostly associated with mild and asymptomatic samples, respectively. Mutation 37(L→F) of NSP6 has a similar trend; 31.91% mild samples and 63.33% of asymptomatic samples showed 37(L→F) mutation, whereas only 1.35% deceased samples had a mutation at 37(L→F). On the contrary, a mutation at 323(P→L) of NSP12 was present in 93.24% of the deceased samples; 51.06% of mild samples and 23.33% of asymptomatic samples have 323(P→L) mutations. Another major mutation of NSP12, 97(A→V) is mostly associated with mild (21.28%) and asymptomatic (63.33%) samples. For NSP14, mutations at 177(L→F) are associated only with deceased (8.11%) samples. These were not observed in the asymptomatic and mild type of samples. We did not find patient status data for NSP4 and NSP5 mutations. Since the number of samples having patient status is quite small, to explore the statistical significance of our observations, we performed Fisher’s exact test. The mutations having *p*-value ≤ 0.05 in Fisher’s exact test are listed in Supplementary Table S1 along with their significance level.

### Structural Analysis of the Wild-Type and Mutant Non-structural Proteins

**FIGURE 3:**
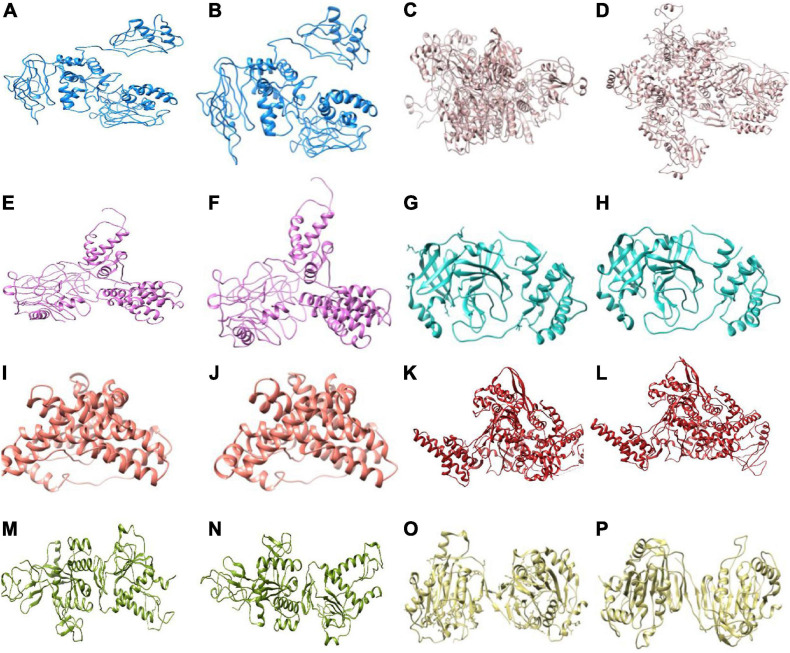
3D structures (shown in cartoon representation) of the most frequently mutated non-structural proteins (NSPs) along with their wild-type (WT) structure. **(A)** NSP2(WT), **(B)** NSP2[496(Q→P)], **(C)** NSP3(WT), **(D)** NSP3[994(A→V)], **(E)** NSP4(WT), **(F)** NSP4[380(A→V)], **(G)** NSP5(WT), **(H)** NSP5[254(S→F)], **(I)** NSP6(WT), **(J)** NSP6[37(L→F)], **(K)** NSP12(WT), **(L)** NSP12[323(P→L)], **(M)** NSP14(WT), **(N)** NSP14[177(L→F)], **(O)** NSP16(WT), and **(P)** NSP16[298(N→L)].

Three-dimensional model structures of 5 WT NSPs and 36 mutant NSPs extracted from Indian patients were generated, and their structural validations were done using various structure validation tools (Table 1). 3D structures were modeled via homology modeling approach using the MODELER program (Webb and Sali, 2016). WT NSP models collected from the Zhang lab COVID-19 resource are also displayed for comparison purposes (Zhang Lab, 2020). 3D coordinates of these models are made available via the DbNSP InC database, and the corresponding links are provided under the “3D/Tertiary structure analysis” analysis column. Figure 3 shows the structures of the most frequently mutated NSP proteins along with their WT structures.

**TABLE 1:** Information of homology models/crystal structures of frequently mutated (mutation frequency ł2.5% in India) NSPs and corresponding wild type NSPs.

**NSP**	**Mutation**	**Sequence length**	**Template/crystal structure pdb id**	**Verify3D (%)** (Eisenberg et al., 1997)	**ERRAT (%)** (Colovos and Yeates, 1993)	**QMEAN** (Benkert et al., 2011)
NSP2	Wild type^*a*^	1–638	NA	76.96	42.05	−13.1
	496(Q→P)	1–638	Wild type	79.47	34.92	−12.34
NSP3	Wild type^*a*^	1–1945	NA	NA	50.50	−9.46
	994(A→D)	1–1945	Wild type	NA	42.87	−8.71
	1198(T→K)	1–1945	Wild type	NA	43.44	−9.03
NSP4	Wild type^*a*^	1–500	NA	72.60	49.06	−10.88
	380(A→V)	1–500	Wild type	80.20	42.87	−10.58
NSP5	Wild type^*b*^	1–306	6w63	93.11	97.24	0.30
	254(S→F)	1–306	6w63	91.83	93.96	−0.74
NSP6	Wild type	1–290	*ab initio*	83.10	96.44	−2.1
	37(L→F)	1–290	Wild type	87.93	90.78	−2.45
NSP12	Wild type^*b*^	1–932	6yyt	87.34	96.70	−1.53
	97(A→V)	1–932	6yyt	85.87	73.6	−2.19
	323(P→L)	1–932	6yyt	88.1	75.95	−1.98
NSP14	Wild type^*b*^	1–527	5c8t	85.39	62.28	−2.6
	177(L→F)	1–527	Wild type	88.05	55.23	−2.91
NSP16	Wild type^*b*^	1–298	6w75	76.06	90.95	−0.79
	298(N→L)	1–298	Wild type	96.31	84.43	−1.65

**^*a*^Model structure is adopted from Zhang Lab (2020). ^*b*^Crystal structure.**

### Viral–Host Protein–Protein Interaction Network Analysis

**FIGURE 4:**
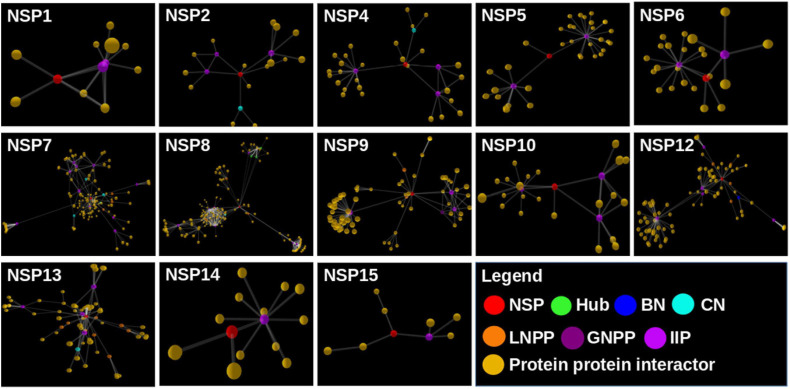
Network view of the interactome of non-structural proteins (NSPs) with their human interactor proteins and their first layer of interactors. Different topologically important nodes (TINs) are marked in different colors. Red, NSPs; yellow, protein–protein interactors; green, hubs; blue, bottlenecks; cyan, central proteins; orange, local network perturbing protein (LNPP); purple, global network perturbing proteins (GNPPs); magenta, important interacting proteins (IIPs).

We found a total of 802 human interactor proteins for 15 NSPs. The viral–host PPIN was constructed for each NSP to identify TINs/proteins, namely, hubs, CNs (Bhattacharyya and Chakrabarti, 2015), BNs (Yu et al., 2007), GNPPs, and LNPPs (Bhattacharyya and Chakrabarti, 2015). Further, important interacting proteins (IIPs) were identified using overlap among any two TINs as described in our earlier report (Bhattacharyya and Chakrabarti, 2015). Table 2 shows the number of IIPs extracted from the SARS-CoV2 and human PPIN. These IIPs may play crucial roles in mediating viral–human interactions. The network representation of these important proteins is displayed in an interactive 3D network viewer at the DbNSP InC database for each NSP. Figure 4 shows the network for NSPs where different TINs are marked in different colors.

**TABLE 2:** Number of important interacting proteins (IIPs) for each NSP-human protein interaction network.

**NSP**	**Number of interactors**	**Number of IIPs**
NSP1	12	3
NSP2	20	4
NSP4	33	4
NSP5	37	3
NSP6	25	3
NSP7	133	11
NSP8	232	8
NSP9	76	4
NSP10	26	4
NSP12	102	6
NSP13	83	5
NSP14	14	2
NSP15	9	2

### Generation of 3D Structures of Viral Non-structural Proteins and Human Interacting Proteins

**FIGURE 5:**
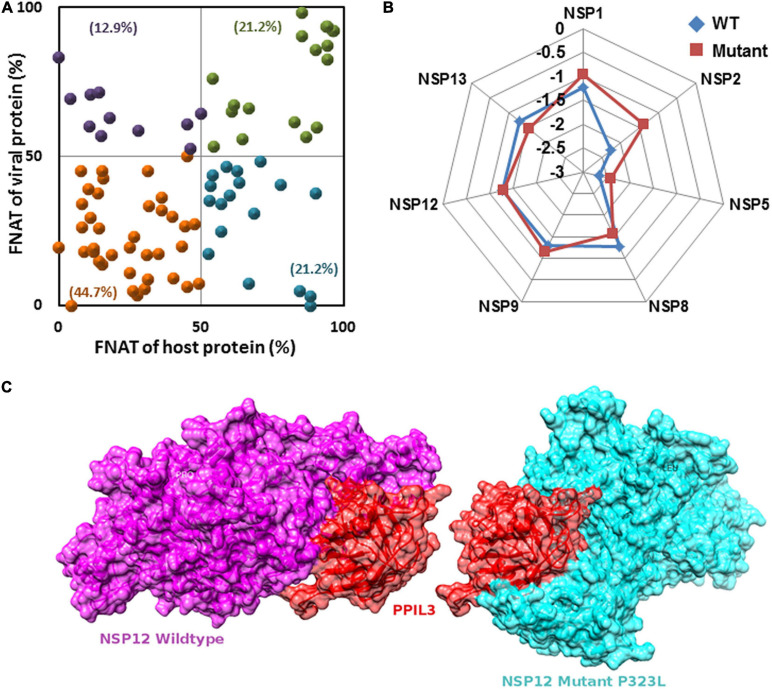
**(A)** Fraction of conserved native contacts (FNATs) of non-structural protein (NSP)–host protein–protein interactome (PPI) complexes formed by the mutant NSPs were calculated with respect to the complexes formed by the wild-type (WT) NSPs. FNAT for both human and viral proteins are plotted. Green, purple, orange, and blue points indicate mutant NSP–human PPI complexes with FNAT values in quadrants I, II, III, and IV, respectively. Percentage values show frequency of each quadrant FNAT complex. **(B)** The percentage of total energy deviation before and after 100 ns of molecular dynamics (MD) simulation in WT and mutants NSP complexed with partner human proteins. **(C)** An example where host protein peptidyl-prolyl isomerase like-3 (PPIL3) (red) has a different binding site for wild-type NSP12 (purple) and mutant (P323L) (cyan) sharing only 18% common residues at the interface.

Extensive protein–protein docking approach implemented via PatchDock program was employed to generate 113 complex structures using 7 known NSP structures and 41 predicted (5 WT and 36 mutant) NSP proteins with 28 human proteins with known structures (Supplementary Table S2). Further, structural and chemical properties of the predicted interfaces such as interface area, free energy of dissociation, presence of hydrogen bond and salt bridges, free energy of formation (ΔG_*int*_), and SE gain (ΔG_*solv*_) were calculated to characterize the interfaces (Supplementary Table S2). Finally, using FNAT-based criteria, we have evaluated the alteration of the interface formed by the mutant NSPs with respect to the WT protein complex. Supplementary Table S2 and Figure 5 show the interfaces that may have altered significantly in complexes formed by the mutant proteins. Almost 45% of the complexes formed by the mutant NSPs show a significant alteration (FNAT ≤50% for both viral and human proteins forming the probable interaction interface) of the binding interface with respect to that formed by their WT counterparts (Figure 5A). Thirty-four percent of the complexes formed by the mutant NSPs show a significant alteration of the interface (FNAT ≤50%) in either viral or human protein partners. However, the complexes formed by the WT and mutant NSPs are found to be energetically stable as shown by relatively low deviation of overall energy of the complexes before and after 100 ns of MD simulations (Figure 5B). Figure 5C shows one of the examples of a significant alteration of the binding interfaces in NSP12 and human interactor protein, peptidyl-prolyl isomerase like-3 (PPIL3), perhaps due to the mutation at position 323(P→L) of NSP12.

### *In silico* Drug Screening With Known Antiviral and Other Drugs Against the Novel Coronavirus 2 Non-structural Proteins

**FIGURE 6:**
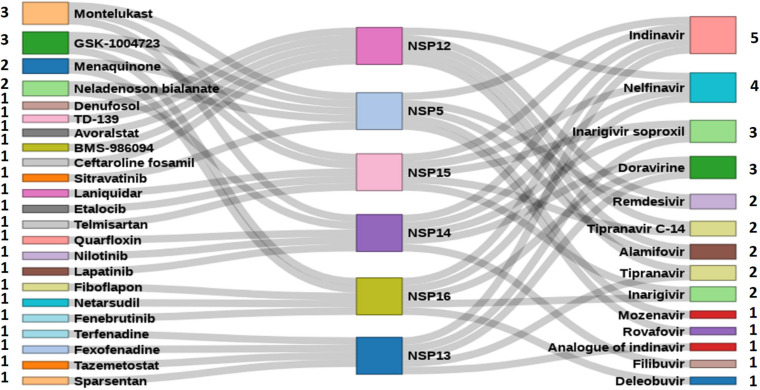
High-throughput virtual screened drugs targeting severe acute respiratory syndrome coronavirus 2 (SARS-CoV2) non-structural proteins (NSPs). Top five antivirals and other known drugs are shown at the right and left sides, respectively, along with the numbers indicating the target NSPs.

**FIGURE 7:**
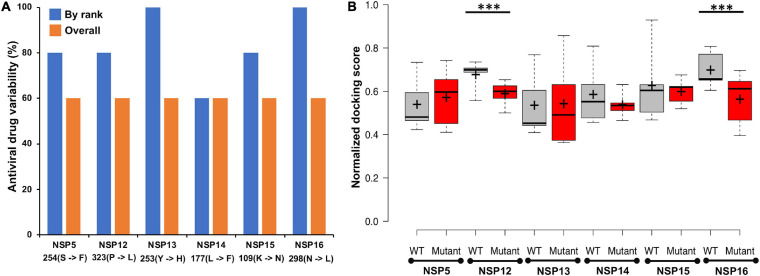
**(A)** The percentage of antiviral drug variability within the best five predicted antivirals against wild-type (WT) and mutant non-structural proteins (NSPs). “By rank” variability was calculated via matching the same drug at the same ranked position within the top five scored drugs, whereas “overall” match was calculated by finding the commonly appearing antiviral drugs within the top five predicted drugs against WT and mutant, respectively. **(B)** The boxplot representation of the docking score of top five ranked antiviral drugs against the wild-type and mutant NSPs, respectively. Median and mean of the scores are shown as line (–) and plus sign (+), respectively. Statistically significant differences (*p*-val ≤0.01) of docking scores are marked with ***.

A total of 111 antiviral compounds and 8,736 known drugs and/or drug-like substances available in the DrugBank (2020) were screened against the NSP WT structures using the GOLD docking software (Jones et al., 1997) where all the fitness scores (GoldScore, ChemPLP, Chemscore, and ASP) were implemented. Compounds commonly appearing (at least in three scoring schemes) among the top 25% solutions of each fitness score were considered as probable inhibitors and were further ranked based on the average value of normalized fitness scores. Figure 6 shows the top five antiviral and known drugs that are likely to act as inhibitors for the SARS-CoV2 NSPs. Several antivirals such as indinavir, nelfinavir, inarigivir soproxil, and doravirine were found to be targeting multiple NSPs. Similarly, known drugs like montelukast and GSK-1004723 seem to bind three or more NSPs as probable targets. Interestingly, the types of antiviral drugs and their relative ranks based on the normalized docking score changed significantly with respect to the WT when the screening was performed against the most frequent mutants of the targeted NSPs {NSP5[254(S→F)], NSP12[323(P→L)], NSP13[253(Y→H)], NSP14[177(L→F)], NSP15[109(K→N)], and NSP16[298(N→L)]} (Figure 7). These findings indicate that drug sensitivity can get altered due to the mutations in the NSPs.

**FIGURE 8:**
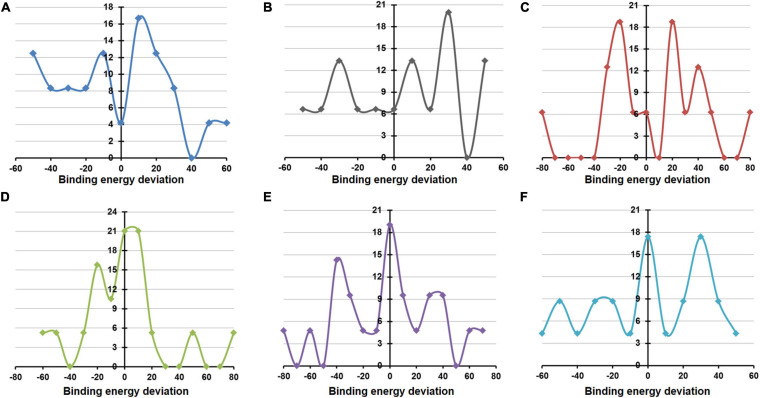
Deviation in binding/docking free energy between pre and post simulation derived drug–non-structural protein (NSP) complexes. **(A–F)** The fraction of screened drugs and their average binding energy deviation for NSP5, NSP12, NSP13, NSP14, NSP15, and NSP16. The *X*-axis represents the percentage of average binding energy deviation, and the *Y*-axis represents the fraction of screened molecules.

MD simulations implemented by GROMACS were also undertaken to evaluate the structural and energetic stabilities of the drug–NSP complexes retrieved from the molecular docking-based screening procedure. Drug–NSP complexes with progressive stabilized binding free energy profiles suggest better stability. Figure 7 shows higher a fraction of the WT complexes that remain stable (±20% deviation) or getting more stable (>20% deviation) in terms of binding free energy throughout the duration of the simulation. For most of the NSPs, the highest peaks observed either for no deviation or at positive binding energy deviation ranges indicate the stability of the complexes (Figure 8).

### Molecular Dynamics Analysis

**FIGURE 9:**
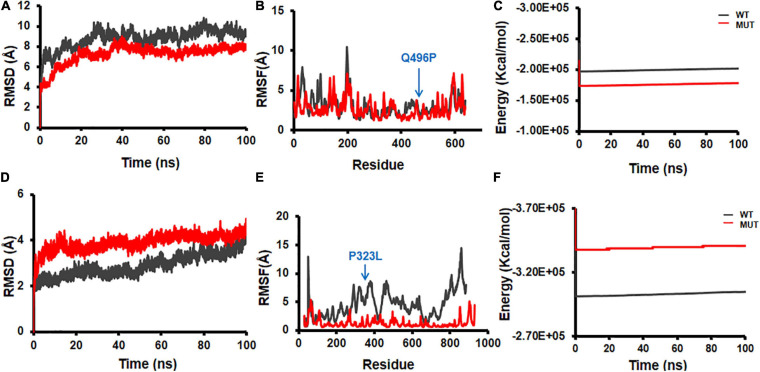
Molecular dynamics (MD) simulation of wild-type (WT) and mutant structures. **(A)** Root mean square deviation (RMSD) plot, **(B)** root mean square fluctuation (RMSF) plot, and **(C)** energy plot for NSP2 WT and 496(Q→P) mutant. **(D)** RMSD plot, **(E)** RMSF plot, and **(F)** energy plot for complex of NSP12 and 323(P→L) mutant. The mutant sites are marked by blue arrows in **(B,E)**.

Structural flexibilities represented by RMSD and RMSF of the WT and mutant NSPs were calculated and compared to evaluate the probable structural and functional alterations that might be due to the mutations. The current version of DbNSP InC provides MD results of WT and mutated NSP1, NSP2, NSP5, NSP8, and NSP12. Figure 9 shows the RMSD, RMSF, and energy profiles of selected mutants from NSP2 and NSP12 as examples to demonstrate marked variations with respect to their WT counterparts. For NSP2, a mutation at 496(Q→P) resulted in lower RMSD (Figure 9A) and higher energy (Figure 9C), whereas RMSF remains almost equally fluctuating compared with WT NSP2 (Figure 9B). For the most prevalent mutation in India, 323(P→L) of NSP12, RMSD has increased (Figure 9D), RMSF (Figure 9E) has reduced significantly, and energy has reduced (Figure 9F) compared with those in the WT variant. It indicates that 323(P→L) is likely to be a stable mutation for NSP12.

**FIGURE 10:**
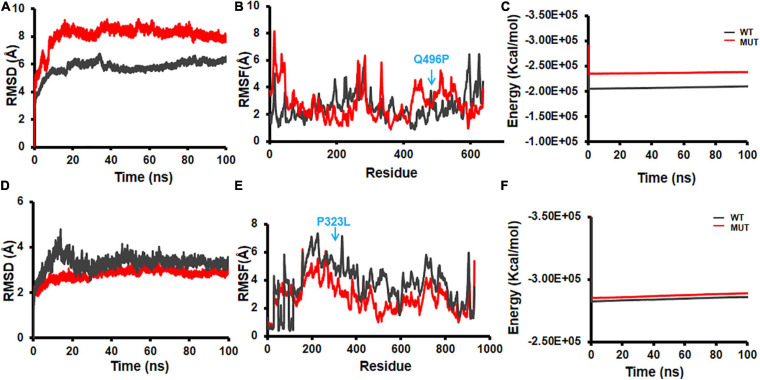
Molecular dynamics (MD) simulation of wild-type and mutant non-structural protein (NSP) complex with human interactor proteins. **(A)** Root mean square deviation (RMSD) plot, **(B)** root mean square fluctuation (RMSF) plot, and **(C)** energy plot for complex of NSP2 with EIF4E2. **(D)** RMSD plot, **(E)** RMSF plot, and **(F)** energy plot for complex of NSP12 with peptidyl-prolyl isomerase like-3 (PPIL3). The mutant site is marked by arrows in **(B,E)**.

Similarly, viral–human protein complexes were also undertaken for MD simulations, and the energy profiles of the complexes during the simulation run were compared between selected mutants and their respective WT NSPs. The current version of DbNSP InC provides MD results of complexes of WT and mutant NSP1, NSP2, NSP4, NSP5, NSP9, NSP12, and NSP13. For each NSP, a complex with one human interactor protein was simulated. The interactor protein was selected based on their topological importance in the corresponding interactome network. Figure 10 shows the representative data for NSP2 and NSP12. For WT and mutant 496(Q→P) complex of NSP2 with human protein EIF4E2, RMSD (Figure 10A), RMSF (Figure 10B), and energy variation (Figure 10C) are shown. EIF4E2 is known to be associated with interferon gamma signaling and innate immune system pathways (Stelzer et al., 2016). In the interactome of NSP2, EIF4E2 appears as an IIP, indicating its topological significance. The binding of EIF4E2 with NSP2 may disrupt the immune response of host. The EIF4E2–NSP2 complex is being targeted by zotatifin drug and is under clinical trial (Yoshimoto, 2020). Supplementary Table S2 shows that EIF4E2 complex has a lower average docking score with mutant NSP2 [496(Q→P)] compared with WT complex. The RMSD profile (Figure 10A) shows that the mutant complex is less stable than the WT complex. Although 496(Q→P) mutation results in slightly lower energy (more stable from) (Figure 9C), binding of EIF4E2 makes the complex less energetically favorable (Figure 10C) than their respective WT counterparts.

Figures 10D–F show the outcomes of MD simulation for complex of WT NSP12 and mutant 323(P→L) with human protein PPIL3. PPIL3, a protein coding gene, helps in protein-folding events (Stelzer et al., 2016) and appears as an IIP in the interactome network of NSP12 (Figure 4). Figure 10D shows that PPIL3 has a stable complex with the mutated structure of NSP12 compared with the WT structure. Supplementary Table S2 also shows that the mutant complex has a higher average docking score compared with the WT structure, while RMSFs are quite less for most of the residues (Figure 10E). The favored association of PPIL3 with most prevalent mutant variation of NSP12 may disrupt the protein-folding mechanisms of host.

## Discussion

COVID-19 disease has caused an unprecedented pandemic, affecting millions around the globe in manifolds. A complete understanding of the underlying virus, SARS-CoV2, is an utmost necessity. Compared with the source samples from Wuhan, SARS-CoV2 has already demonstrated several mutations across the globe, and the mutations are often region specific (Khan et al., 2020; Mercatelli and Giorgi, 2020). In this context, we concentrate on the Indian variants of SARS-CoV2 genomes. The major part of the SARS-CoV2 genome consists of a poly-protein, which comprises 16 NSPs. Our database, DbNSP InC, is dedicated to holistic studies of NSPs of SARS-CoV2 virus obtained from samples collected from different places of India. It showcases the mutational variations of SARS-CoV2 virus along with the impact of the mutations in different aspects including disease severity and spread in different Indian states. This database provides a pool of combinatorial information regarding the probable impact of the mutations on structural and energetic stabilities of the viral NSPs and subsequently on host protein interaction. Moreover, it also provides critical and useful information about the probable antivirals and known drugs that could be testified for development of effective drugs against the novel coronavirus 2 (nCoV2) virus. We are hopeful that DbNSP InC database will be a very useful repository to understand the nature of the nCoV2 variants that prevailed in India and their probable impact on the patho-physiology of the disease.

Over the last 1 year, numerous works have been performed to characterize the SARS-CoV2 proteins and the associated mutations. Several databases and online resources have been developed to aid the fight against the deadly COVID-19 pandemic. Databases like EpiCoV^TM^ platform from GISAID (GISAID, 2020), NCBI-SARS-CoV2 resources (NCBI-SARS-CoV2 Resources, 2020), COVID-19 data portal (EMBL-EBI, 2020), Virus Pathogen Database and Analysis Resource (ViPR) (Pickett et al., 2012), GESS (Fang et al., 2021), CovDB (Zhu et al., 2020), and ViruSurf (Canakoglu et al., 2021) systematically categorized thousands of nCoV2 genome sequences deposited from all over the world. Similarly, resources like Cov3D (Gowthaman et al., 2021), SWISS-MODEL SARS-CoV2 portal (Swiss-Model, 2020), and Zhang lab COVID-19 resource (Zhang Lab, 2020) developed 3D models for SARS-CoV2 proteins for structural characterizations, whereas exhaustive experimental characterization of host protein interactions was revealed by works from Gordon et al. (2020a, b). In addition, countless efforts have been put forward using *in silico* drug screening approaches to identify potential inhibitors of the SARS-CoV2 proteins. Some of the works from India also highlighted the genomic diversity and the phylogenetic profiles of the prevalent strains in the country (Banu et al., 2020; Thakur et al., 2020; INDICOV, 2021; Jain et al., 2021; Phylovis, 2021). However, most of these works are discrete in nature, and a combined unified effort characterizing a country- or region-specific mutational profile of the SARS-CoV2 proteins, especially for the NSPs, is warranted. DbNSP InC aims to encompass the country- and state-specific mutational profile of the prevalent SARS-CoV2 genomes and to further provide a comprehensive characterization of the frequently observed mutations in terms of the probable impacts on their structure, function, and interactions with host proteins and target small molecule inhibitors. To the best of our knowledge, this kind of large-scale, multilevel characterization of country (India) specific SARS-CoV2 NSP mutational analysis followed by estimation of the probable impact of the mutant proteins has not been reported before.

The mutation analysis of the NSP sequences of SARS-CoV2 virus collected from Indian patients reveals several mutations that were not observed in the samples collected in Wuhan, China, from where the virus spread by human contact. Also, some mutations, which are frequently observed in the Wuhan samples, were not observed in the Indian samples. It seems that NSP12 (RdRp) is the most changing protein among the NSPs found in the Indian population. The mutation at site 323 of NSP12 is caused by change of amino acid from P to L. This mutation was observed in 78.44% samples. Moreover, this mutation was observed in 93.24% of samples where patients did not survive. It implies that 323(P→L) mutation of NSP12 is the most lethal mutation among all mutations of all NSPs. From the PAM250 substitution matrix, the score of P→L transition is -3, indicating strong dissimilarity between the mutated and reference sequences. However, 323(P→L) mutation of NSP12 is not unique to the Indian samples. Although not observed in the Wuhan samples, its occurrence is already reported as prevalent in European countries and also in North America (Kannan et al., 2020; Pachetti et al., 2020). This mutation also has a prevalence of a co-occurrence with other mutations (Pachetti et al., 2020). NSP12 creates the core polymerase complex with NSP7 and NSP8 (Hillen et al., 2020; Peng et al., 2020; Wang et al., 2020), and site 323 locates near the binding interface of NSP8 and NSP12 (Hillen et al., 2020). The proline (P) amino acid creates hydrogen bond with NSP8 (Mutlu et al., 2020). The P→L mutation is preferable to NSP8–NSP12 binding and thus promotes viral replication (Kannan et al., 2020). Hence, the role of 323(P→L) mutation needs attention while designing antiviral drugs targeting the polymerase complex. Moreover, 323(P→L) of NSP12 has a strong co-occurrence with spike protein mutation at 614(D→G) worldwide (Kannan et al., 2020). Supplementary Figure S3 illustrates the co-occurrence of 323(P→L) and 614(D→G) in the Indian samples also. 323(P→L) is also known to co-occur with 241(C→U) mutation of 5′-UTR of SARS-CoV2 (Kannan et al., 2020). These co-occurrences perhaps enhance the viral activity, making it lethal for human survival. The other mutation 97(A→V) of NSP12 appeared in Singapore, Malaysia, and Europe (GISAID, 2020). 1198(T→K) mutation of NSP3 is prevalent in Asian countries, such as Singapore Malaysia, and also in the United Kingdom (GISAID, 2020). 37(L→F) mutation of NSP6 is also observed in other countries including in samples from Wuhan, China (Figure 1A and Supplementary Figure S1). It reduces the stability of the protein structure (Benvenuto et al., 2020; Mercatelli and Giorgi, 2020). Hence, this mutation appears favorable to human beings, and also, it is not associated with deceased samples (Figure 1C). We also compared the frequencies of the most frequent mutations in India in the global scenario. Supplementary Figure S4 compares the frequencies of the mutations shown in Figure 1A in different continents. Here, Asian data are considered, excluding India data. We observed 323(P→L) mutation of NSP12 across the globe. Mutation 37(L→F) of NSP6 is also observed in different continents but more frequently in India and Asia. Mutations 97(A→V) of NSP12 and 1198(T→K) of NSP3 appear specific to India and Asia. Mutation 994(A→D) of NSP3 emerges as specific to India.

Depending on the availability, the crystal structures and/or 3D models of WTs and mutated NSPs are listed in the DbNSP InC database. The crystal structures are available for WTs NSP5, NSP7, NSP9, NSP10, NSP12, NSP15, and NSP16. We have constructed 3D model structures of WTs NSP1, NSP6, NSP8, NSP13, and NSP14 by homology modeling, and we further validated them using multiple structure validation tools. 3D models retrieved from the Zhang Lab (2020) are also shared for comparison purposes. In general, validation scores of our models are comparable and/or better than those obtained from the Zhang lab models. We observed for NSP1 that QMEAN and Verify3D scores are better for our model than the corresponding scores from Zhang lab NSP1 model, whereas our model has a lower ERRAT score. For NSP6, our model obtained better scores for all the validation methods, whereas for NSP8, ProSA z-score and ERRAT quality factor are comparable with those of the Zhang lab. For NSP13, the QMEAN score is better, but Verify3D and ERRAT scores are not compared with that achieved from the Zhang lab-derived model. Verify3D and QMEAN scores are better for our NSP14 model. However, we have listed the WT model structures NSP2, NSP3, and NSP4 obtained from the Zhang lab in our DbNSP InC database. Based on the crystal and modeled structures of WT NSPs, 36 mutant model structures were generated. All these 3D models were evaluated using various structure validation tools such as PROCHECK (Zhang Lab, 2020), ERRAT (Laskowski et al., 1993), Verify 3D (Colovos and Yeates, 1993), QMEAN (Eisenberg et al., 1997), and ProSA (Benkert et al., 2011). The validation scores of these mutant models are comparable and/or better than those of the WT counterparts. This advocates their comparable stability and utilization of these mutant structures in downstream analyses of protein–protein interaction as well as protein–drug interactions.

We further constructed interactome for each NSP with their human host proteins, along with their first layer of interactors. The virus–host protein interactome is necessary for understanding how the virus proteins interact with human immune systems and proteins involved in various biological pathways (Perrin-Cocon et al., 2020). We observed that NSP8 has the highest number of interactors, 232, followed by NSP7, which has 133 interactors (Table 2). NSP7 interactome produced the highest number of IIPs, 11, followed by NSP8, which has 8 IIPs. Overall, 59 IIPs were identified out of 802 human interactor proteins for 15 NSPs. A composite interactome involving all 15 NSPs and their 802 human interactors (first layer) were also created to examine the interconnectivity between them where only NSP10 and NSP6 interactomes were found to be disjointed (Supplementary Figure S5). Guided by the interactome analysis, we generated 113 complex structures using 48 (WT and mutant) NSP and 28 human proteins. Further, structural and chemical properties calculated from the predicted interfaces have shown significant alterations of the interface formed by the mutant NSPs with respect to the WT protein complex. These findings may provide mechanistic insight toward differential host interaction pattern of the variants NSPs, which could relate to varied host responses of the patients infected with the variant nCoV2 virus. However, these preliminary analyses need to be verified by in-depth experimental studies to establish altered interaction and its connections to the patho-physiology of the disease. Nevertheless, our findings on host protein interactions provide clues and direction to future in-depth analyses of specific viral–host protein interaction studies.

A total of 111 antiviral and 8,736 known drugs were screened against various enzymes (NSP5, NSP12, NSP13, NSP14, NSP15, and NSP16) of SARS-CoV2 using a rigorous HTVS procedure to identify the probable candidate that can act against SARS-CoV2 NSP enzymes. Several drug candidates have been identified that can act on multiple targets (Figure 6). The antiviral drug indinavir is targeting five SARS-CoV2 enzymes (NSP5, NSP13, NSP14, NSP15, and NSP16). Indinavir is a known HIV-1 protease inhibitor (Lv et al., 2015). Some of these antivirals (e.g., remdesivir, nelfinavir, and tipranavir) are part of ongoing clinical trials (ASHP, 2021), whereas drugs like nilotinib, lapatinib, indinavir, nelfinavir, tipranavir, montelukast, and telmisartan are also reported as potential inhibitors of NSPs (Ghahremanpour et al., 2020). Nelfinavir has also been identified as a SARS-CoV2 protease inhibitor by supervised MD simulation (Bolcato et al., 2020). It also appears as a drug effective in saving SARS-CoV2-affected cells from death (Ianevski et al., 2020; Musarrat et al., 2020). Similarly, other antiviral drugs like doravirine, alamifovir, inarigivir, and inarigivir soproxil were found to target multiple targets. Among the drug bank drugs, montelukast targets three NSPs. Montelukast has anti-inflammatory effects, reduces oxidative stress, and appears as a potential treatment of COVID-19 (Fidan and Aydoğdu, 2020). It is currently being used in a clinical trial (Clinical Trials Gov, 2020). The other known drugs neladenoson bialanate and menaquinone were also found to act against multiple SARS-CoV2 enzymes. Menaquinone (vitamin K2) deficiency may lead to severity for SARS-CoV2-infected patients and appears as a supplementary in reducing COVID-19 mortality rate (Berenjian and Sarabadani, 2020). These multi-target drugs can be efficient drug candidates against SARS-CoV2. However, screening against the mutant forms of the NSPs yielded quite different antiviral drug populations, at least within the top five ranked antivirals selected based on the normalized composite docking scores (Figure 7). This finding is exciting and indicates a probable alteration of drug sensitivity of the NSPs due to the acquired mutations. However, further in-depth testing is required to confirm the likelihood of the effective alteration of drug sensitivity. Several studies have been reported in the past few months involving drug screening against SARS-CoV2 proteins. However, to the best of our knowledge, our study is one of the few (Swiss-Model, 2020; Gowthaman et al., 2021) to screen both antivirals and other known drugs against all six WT and mutant NSPs (NSP5, NSP12, NSP13, NSP14, NSP15, and NSP16) together. This composite HTVS provides a uniform perspective and platform for shortlisting drugs that could be further testified via in-depth cell free and cell-based assays. Drug repurposing with approved or investigational drugs is perhaps the most effective, rational, and timely strategy for identification of effective drugs against COVID-19. We believe that our findings, which have been made freely available through DbNSP InC, will help the community to attest to the effectiveness of some of the top-scoring drugs.

We have further complimented our molecular modeling and docking analyses with rigorous, atomistic, and solvent-implicit MD simulations. Atomic-level MD simulations offer a computational route toward characterizing both structural and energetic stabilities of protein–protein as well as protein–ligand complexes. In the absence of sufficient experimental information regarding the host protein and drug binding properties of the SARS-CoV2 NSPs, we utilized MD simulations to characterize and evaluate the predictive docking complexes formed by the WT and mutants. Findings from the MD simulation studies suggest acceptable structural and energetic stabilities of the 3D models as well as protein–protein complexes formed by them. Similarly, our MD simulations using the drug–NSP complexes retrieved from the molecular docking-based screening procedure provide additional screening and filtering criteria for selection of the most likely drug candidates. Drug–NSP complexes with progressive stabilized binding free energy profiles suggest better stability and hence can be used as a selection tool. Our MD analyses with drug–NSP complexes show that a higher fraction of the complexes remains stable (±20% deviation) or becomes more stable (>20% deviation) in terms of binding free energy throughout the duration of the simulation. This would definitely aid current and future drug discovery and re-purposing efforts against COVID-19.

## Conclusion

In conclusion, DbNSP InC emerges as a platform where researchers can get updated information on NSPs of SARS-CoV2 specific to Indian patients. Since many of the mutations, reported in our manuscript as well as provided in DbNSP InC, are observed globally, the corresponding analysis bears relevance even in the global context. In the future, we will enrich DbNSP InC by including more information obtained via structure analysis, host protein interaction, MD simulation, and drug screening. The database will also be updated regularly with the availability of newer sequencing and mutational data.

## Data Availability Statement

The original contributions presented in the study are included in the article/Supplementary Material, further inquiries can be directed to the corresponding author/s.

## Author Contributions

NB performed all the sequence analyses and mutational and phylogenetic analyses. KK performed the drug screening and MD analysis. PM and SD performed the protein–protein interaction study. IK, SB, and AC performed all the modeling and partial MD analysis. NB and SC wrote the manuscript and conceptualized and coordinated the project. All authors contributed to the article and approved the submitted version.

## Conflict of Interest

The authors declare that the research was conducted in the absence of any commercial or financial relationships that could be construed as a potential conflict of interest.
