# Supplementary material for: Structural and Drug Screening Analysis of the Non-structural Proteins of Severe Acute Respiratory Syndrome Coronavirus 2 Virus Extracted From Indian Coronavirus Disease 2019 Patients
Source: Front Genet. 2021 Mar 9;12:626642. doi: 10.3389/fgene.2021.626642 (PMC7985531; doi:10.3389/fgene.2021.626642)
Supplement: Supplementary file 1 [file Data_Sheet_1.docx]

**Supplementary information**

**Structural and Drug Screening Analysis of the Non-structural Proteins of Severe Acute Respiratory
Syndrome Coronavirus 2 Virus Extracted From Indian Coronavirus Disease 2019 Patients**

Nupur Biswas^*^, Krishna Kumar, Priyanka Mallick, Subhrangshu Das, Izaz Monir Kamal, Sarpita Bose, Anindita Choudhury and Saikat Chakrabarti^*^

Structural Biology and Bioinformatics Division, Council for Scientific and Industrial Research (CSIR) - Indian Institute of Chemical Biology (IICB), Kolkata, West Bengal, India

**Corresponding authors*

E-mail: nupur@csiriicb.res.in, saikat@iicb.res.in


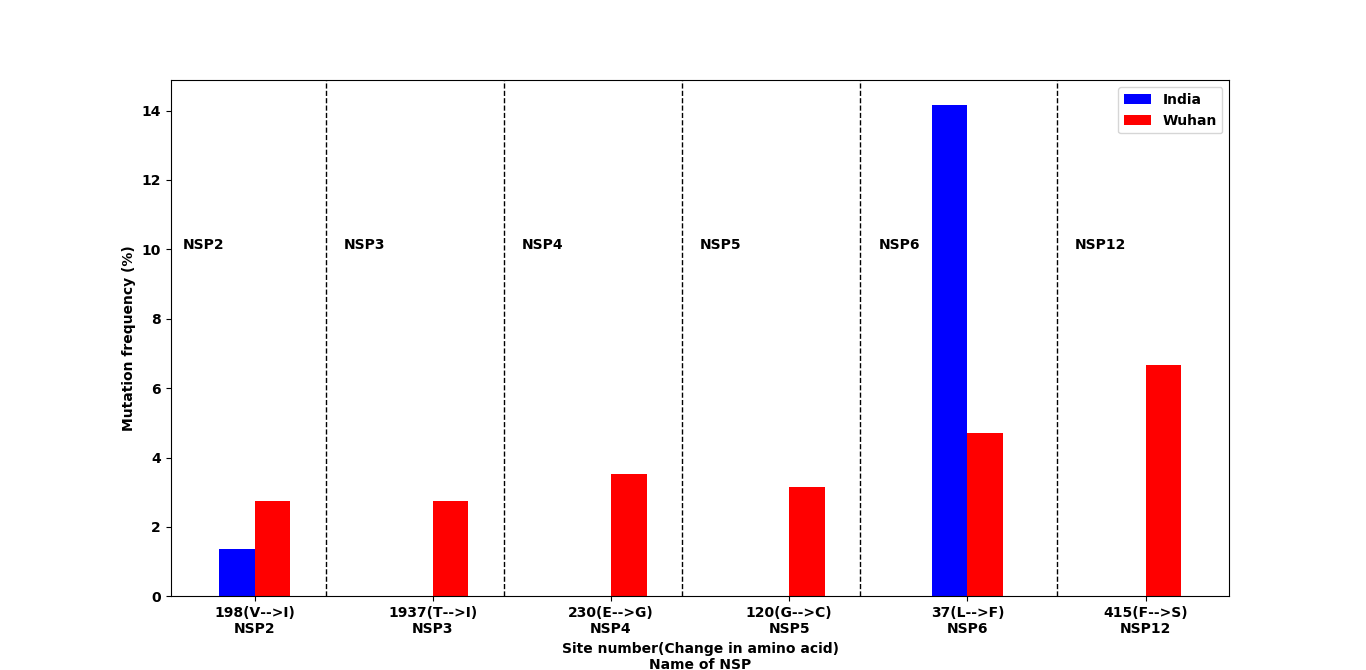


**Figure S1:** Comparison of mutation frequencies for different NSPs from samples collected from India (blue) and Wuhan, China (red) where frequencies ≥ 2.5% in Wuhan. Dashed lines are drawn to separate NSPs. This image is constructed considering only full length sequences.


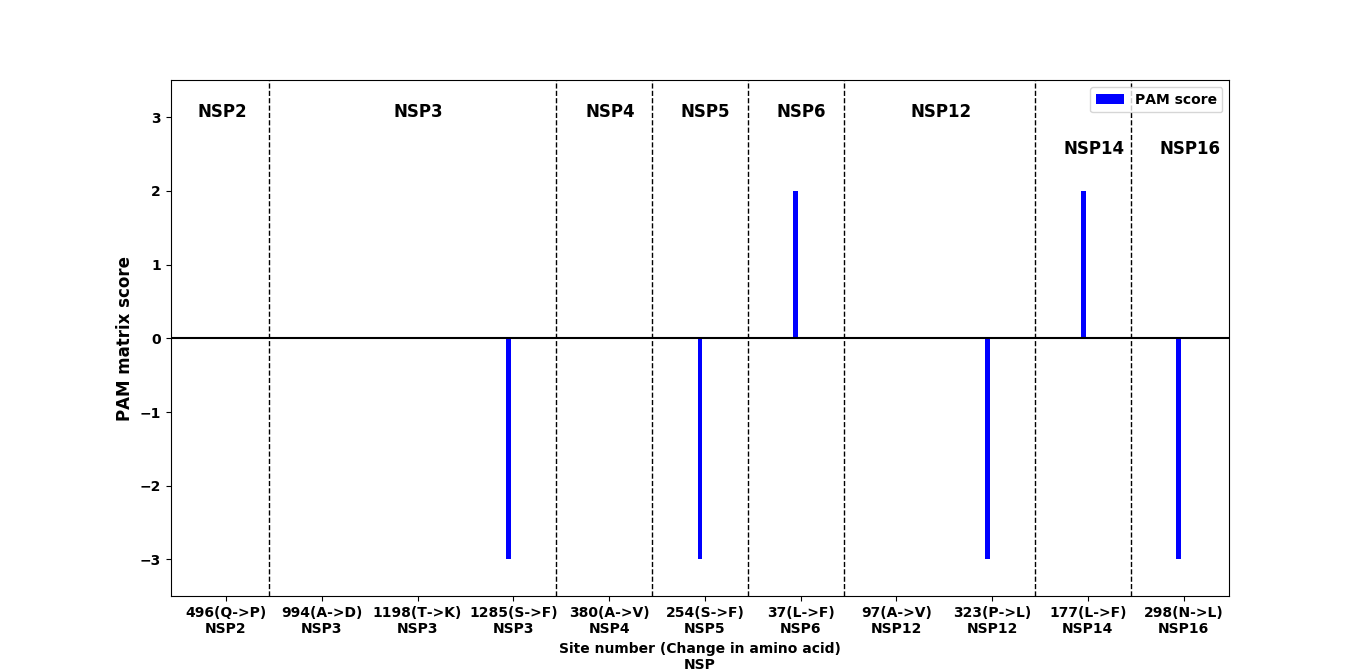


**Figure S2:** PAM 250 matrix scores of mutations frequently observed in India. Dashed lines are drawn to separate NSPs.


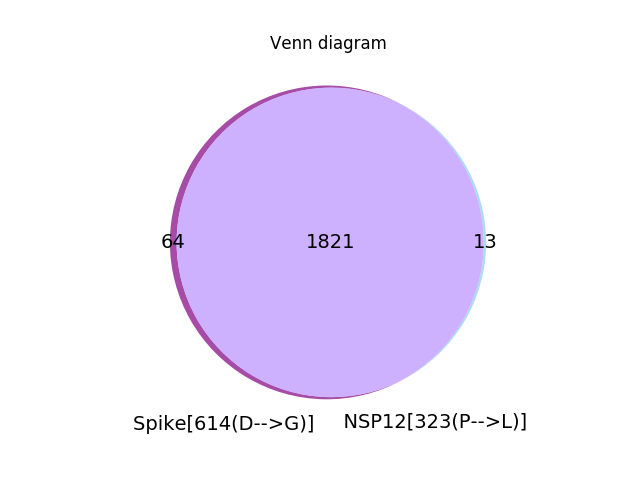


**Figure S3:** Venn diagram showing co-occurrence of 614 (D🡪G) mutation of spike protein with 323 (P🡪L) mutation of NSP12 in Indian samples.


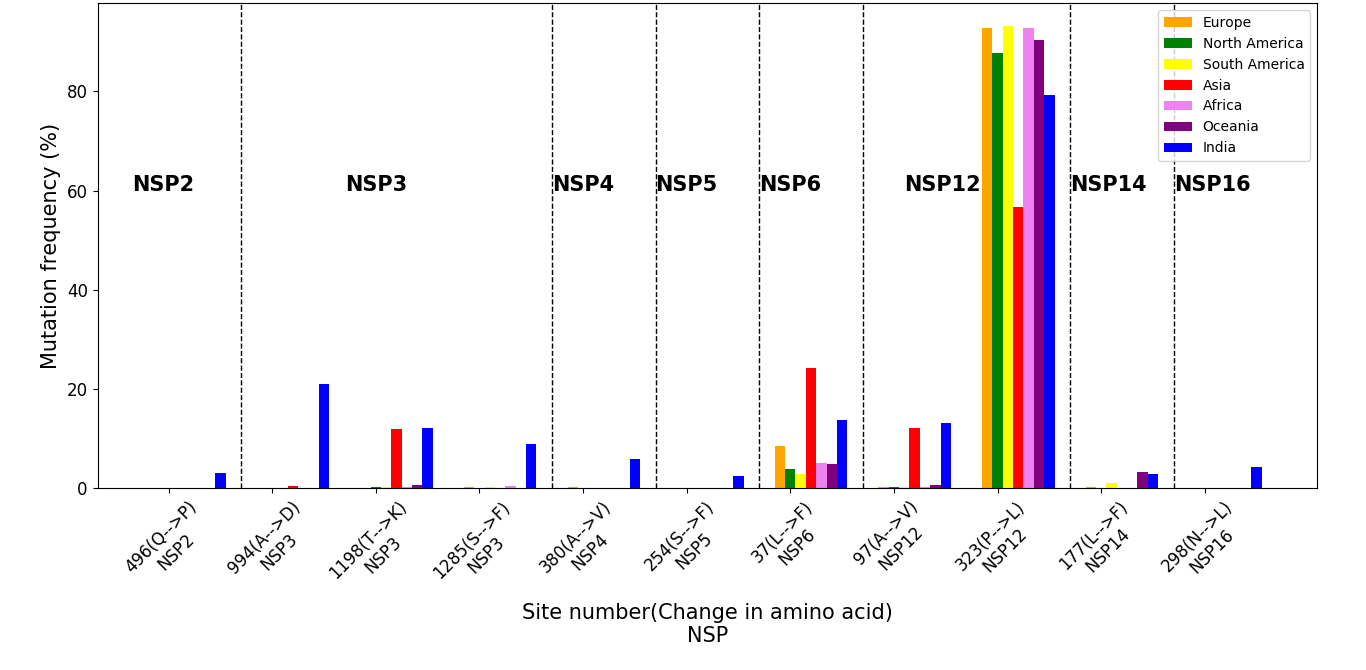


**Figure S4:** Comparison of mutation frequencies of frequent (≥ 2.5%) mutations of India with respect to the same estimated from different continents. Dashed lines are drawn to separate NSPs.


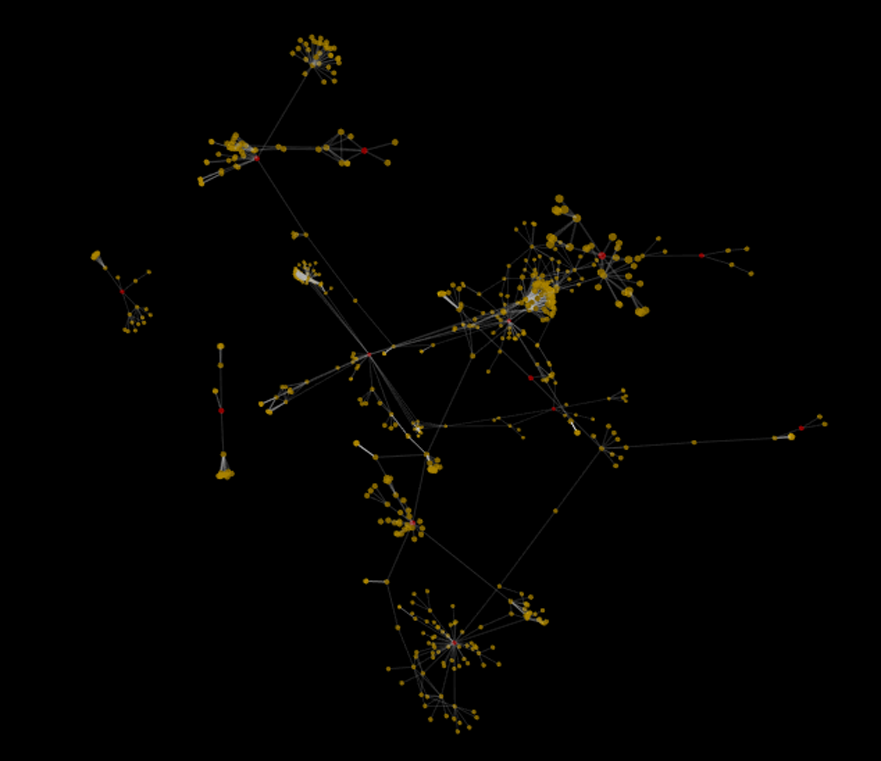


**Figure S5:** Combined interactome network of all NSPs (marked in red) with their human interactor proteins (yellow) along with their first layer of interactors. Disjointed interactomes of NSP10 and NSP6 are placed on the left.

**Table S1:** *p-values* extracted from Fisher exact test for various mutations observed in different patient types. The table shows mutations having *p-value* ≤ 0.05.

| **NSP** | **Mutation site**  **(Change in amino acid)** | **Patient status** | **Significance level**  **(≤ p-value)** |
| --- | --- | --- | --- |
| NSP2 | 496(Q🡪P) | Deceased | ≤ 0.01 |
| NSP3 | 994(A🡪D) | Deceased | ≤ 0.001 |
| NSP3 | 994(A🡪D) | Mild | ≤ 0.05 |
| NSP3 | 1198(T🡪K) | Deceased | ≤ 0.01 |
| NSP3 | 1198(T🡪K) | Mild | ≤ 0.05 |
| NSP3 | 1198(T🡪K) | Asymptomatic | ≤ 0.001 |
| NSP3 | 1285(S🡪F) | Deceased | ≤ 0.05 |
| NSP3 | 1285(S🡪F) | Mild | ≤ 0.01 |
| NSP3 | 1285(S🡪F) | Asymptomatic | ≤ 05 |
| NSP4 | 380(A🡪V) | Deceased | ≤ 0.01 |
| NSP4 | 380(A🡪V) | Mild | ≤ 0.05 |
| NSP6 | 37(L🡪F) | Deceased | ≤ 0.001 |
| NSP6 | 37(L🡪F) | Mild | ≤ 0.001 |
| NSP6 | 37(L🡪F) | Asymptomatic | ≤ 0.001 |
| NSP12 | 97(A🡪V) | Deceased | ≤ 0.01 |
| NSP12 | 97(A🡪V) | Asymptomatic | ≤ 0.001 |
| NSP12 | 323(P🡪L) | Deceased | ≤ 0.001 |
| NSP12 | 323(P🡪L) | Mild | ≤ 0.001 |
| NSP12 | 323(P🡪L) | Asymptomatic | ≤ 0.001 |
| NSP14 | 177(L🡪F) | Deceased | ≤ 0.05 |
| NSP16 | 288(N🡪L) | Deceased | ≤ 0.05 |

**TableS2**: Protein-protein docking and interface properties of NSP-Human interactor wild type and mutant complexes.

| NSP | Human Interactor | Complex Type | Site | PatchDock Result | | PISA Result | | | | FNAT score of Chain1  (host) | FNAT score of Chain2  (virus) |
| --- | --- | --- | --- | --- | --- | --- | --- | --- | --- | --- | --- |
|  |  |  |  | Avg Patchdock Score of largest cluster | Top Patchdock Score of largest Cluster | Interface Area () | No of H-Bond in interface region | No of Salt-Bridge in interface region | Binding Energy (kcal/mol) |  |  |
| NSP1 | POLA1 | Wildtype |  | 15768.53 ± 1044.72 | 19288 | 2233.2 | 14 | 8 | -12.5 | 100 | 100 |
|  |  | Mutant1 | 16 | 15326.23 ± 982.5 | 18850 | 2294.3 | 12 | 2 | -20 | 60 | 37.1 |
|  |  | Mutant2 | 83 | 15015.42 ± 809.71 | 17440 | 2263.7 | 19 | 1 | -15.6 | 53.85 | 43.55 |
|  |  | Mutant3 | 114 | 14978.88 ± 1264.02 | 17700 | 2184.1 | 16 | 4 | -10.4 | 15.38 | 45.16 |
|  |  | Mutant4 | 124 | 15196.38 ± 892.99 | 17020 | 2167.9 | 15 | 5 | -13.3 | 26.15 | 4.84 |
|  |  | Mutant5 | 135 | 15676.5 ± 1038.94 | 18286 | 2262.6 | 11 | 6 | -12.8 | 93.85 | 93.55 |
|  | PRIM1 | Wildtype |  | 13899 ± 1241.97 | 17904 | 1943.1 | 12 | 4 | -18.1 | 100 | 100 |
|  |  | Mutant1 | 16 | 13647.30 ± 799.94 | 15522 | 1774.5 | 8 | 4 | -16.2 | 52.63 | 17.39 |
|  |  | Mutant2 | 83 | 13521.33 ± 486.6 | 14390 | 1539.7 | 4 | 5 | -12.5 | 61.4 | 67.39 |
|  |  | Mutant3 | 114 | 13041.3 ± 851.59 | 15638 | 1940.1 | 16 | 0 | -1.3 | 0 | 19.57 |
|  |  | Mutant4 | 124 | 13685.12 ± 570.96 | 14838 | 1833.6 | 20 | 8 | -2.8 | 35.09 | 17.39 |
|  |  | Mutant5 | 135 | 13764.66 ±767.63 | 15286 | 1500.9 | 18 | 10 | -0.2 | 28.07 | 58.7 |
|  | PRIM2 | Wildtype |  | 14590.33 ± 1119.8 | 18142 | 1985.7 | 16 | 1 | -15 | 100 | 100 |
|  |  | Mutant1 | 16 | 14371.69 ± 1025.31 | 17510 | 1875.4 | 11 | 4 | -15.8 | 85.19 | 90.2 |
|  |  | Mutant2 | 83 | 14067.62 ± 693.27 | 15734 | 1994.2 | 18 | 8 | -6.8 | 11.11 | 29.41 |
|  |  | Mutant3 | 114 | 13846.4 ± 763.19 | 14668 | 1699.4 | 6 | 12 | -2.3 | 14.81 | 56.86 |
|  |  | Mutant4 | 124 | 14580.42 ±1183.91 | 18286 | 2038 | 20 | 3 | -14.3 | 85.19 | 98.04 |
|  |  | Mutant5 | 135 | 14446.61 ± 887.9 | 18014 | 2207.5 | 14 | 5 | -18 | 96.3 | 92.16 |
| NSP2 | EIF4F2 | Wildtype |  | 13,548.46 ±793.28 | 16126 | 3191.9 | 97 | 20 | -15 | 100 | 100 |
|  |  | Mutant1 | 27 | 14,068.30 ±1062.27 | 15208 | 3224.5 | 193 | 7 | -25.7 | 7.95 | 45.28 |
|  |  | Mutant2 | 198 | 13,789.36 ±865.90 | 15600 | 2410 | 141 | 26 | -13.6 | 25 | 19.34 |
|  |  | Mutant3 | 381 | 13901.69 ± 808.56 | 15688 | 2142.3 | 19 | 3 | -12.8 | 60.78 | 65.22 |
|  |  | Mutant4 | 496 | 13422.28 ± 1211.47 | 16090 | 2043.5 | 14 | 3 | -21 | 31.37 | 8.7 |
|  | POR | Wildtype |  | 14,806.18 ±847.32 | 15908 | 2091.8 | 38 | 1 | -10.4 | 100 | 100 |
|  |  | Mutant1 | 27 | 15,097.00 ±844.72 | 17262 | 4648.1 | 131 | 22 | -13.6 | 10.34 | 39.06 |
|  |  | Mutant2 | 198 | 14,734.48 ±696.09 | 15674 | 3253.3 | 74 | 8 | -9.8 | 39.66 | 29.69 |
|  |  | Mutant3 | 381 | 14792.43 ± 942.38 | 16796 | 2273 | 16 | 5 | -19.4 | 47.46 | 27.27 |
|  |  | Mutant4 | 496 | 14772 ± 1092.54 | 14772 | 2335.7 | 20 | 6 | -7.5 | 49.15 | 7.27 |
| NSP4 | IDE | Wildtype |  | 14130.67±1286.91 | 17462 | 3191.2 | 37 | 5 | -14.4 | 100 | 100 |
|  |  | Mutant 1 | 33 | 16521.87±687.54 | 17868 | 2260.7 | 9 | 4 | -19.9 | 43.16 | 20 |
|  |  | Mutant 2 | 279 | 16471.4 ± 1178.2 | 19514 | 2253.3 | 14 | 0 | -13.6 | 31.58 | 33.33 |
|  |  | Mutant 3 | 380 | 16432 ± 989 | 16432 | 2739.5 | 26 | 7 | -7.3 | 64.21 | 56 |
| NSP5 | HDAC2 | Wildtype |  | 13156.76 ± 699.37 | 15426 | 1838.7 | 7 | 0 | -7.5 | 100 | 100 |
|  |  | Mutant1 | 96 | 13326.8 ± 1066.40 | 16670 | 2145.2 | 20 | 11 | -8.8 | 94.12 | 93.65 |
|  |  | Mutant2 | 136 | 13450.09 ± 990.53 | 17064 | 1918.4 | 8 | 2 | -12.1 | 94.12 | 87.3 |
|  |  | Mutant3 | 140 | 13556.36 ± 1027.67 | 15068 | 2066.2 | 18 | 13 | -8.7 | 88.24 | 3.17 |
|  |  | Mutant4 | 142 | 13731.55 ± 1363.97 | 17350 | 2008.6 | 11 | 0 | -11 | 96.08 | 92.06 |
|  |  | Mutant5 | 204 | 13224.62 ± 1235.61 | 15634 | 18907 | 10 | 1 | -11.5 | 90.2 | 85.71 |
|  |  | Mutant6 | 254 | 13757.86 ± 1205.08 | 18308 | 2121 | 14 | 4 | -3.7 | 84.31 | 4.76 |
|  |  | Mutant7 | 284 | 13728.4 ± 1246.95 | 14842 | 1828.7 | 12 | 0 | -6 | 94.12 | 82.54 |
| NSP6 | SIGMAR1 | Wildtype |  | 13628.62 ± 760.44 | 16122 | 1828 | 3 | 0 | -22.4 | 100 | 100 |
|  |  | Mutant 1 | 37 | 13743.48 ± 1216.45 | 16954 | 1553.7 | 6 | 2 | -11.7 | 8.89 | 18 |
| NSP7 | GNB1 | Wildtype |  | 11334.67 ± 1001.87 | 13232 | 1521.2 | 4 | 4 | -16.2 | 100 | 100 |
|  |  | Mutant1 | 50 | 11094.27 ± 1014.5 | 13286 | 1443.3 | 4 | 1 | -15.7 | 86.67 | 56.41 |
|  | RHOA | Wildtype |  | 10720.08 ± 1383.15 | 14754 | 1670.4 | 5 | 3 | -14.1 | 100 | 100 |
|  |  | Mutant1 | 50 | 10239.85 ± 876.28 | 12322 | 1544.4 | 11 | 4 | -11.1 | 90 | 37.5 |
| NSP8 | EXOSC2 | Wildtype |  | 13227.12 ± 917.59 | 14962 | 1662.1 | 5 | 3 | -7.8 | 100 | 100 |
|  |  | Mutant1 | 26 | 13471.2 ± 1501.24 | 16806 | 2115.7 | 14 | 7 | -8.1 | 36.17 | 36.36 |
|  |  | Mutant2 | 37 | 13255.30±1025.80 | 15498 | 1997.6 | 12 | 5 | -15.7 | 58.7 | 46.51 |
|  |  | Mutant3 | 58 | 13645.63±1057.8 | 16092 | 1895.9 | 11 | 0 | -8.7 | 54.35 | 53.49 |
|  |  | Mutant4 | 198 | 13017.6 ± 1057.8 | 15888 | 1946 | 11 | 3 | -10.6 | 36.17 | 31.82 |
|  | EXOSC3 | Wildtype |  | 13100±996.47 | 14856 | 1899.9 | 5 | 4 | -17.4 | 100 | 100 |
|  |  | Mutant1 | 26 | 12716.59 ± 1039.47 | 15416 | 2054.1 | 10 | 2 | -24.8 | 30.77 | 16.92 |
|  |  | Mutant2 | 37 | 12929.64±712.87 | 14012 | 1490.1 | 12 | 0 | -16.9 | 56.86 | 25 |
|  |  | Mutant3 | 58 | 13073.14±971.13 | 15294 | 1949.4 | 12 | 3 | -28.8 | 45.1 | 6.25 |
|  |  | Mutant4 | 198 | 13070.54 ± 1112 | 15914 | 1955.6 | 12 | 4 | -18.8 | 44.23 | 26.69 |
|  | EXOSC5 | Wildtype |  | 12160.5±1149.28 | 15078 | 1833.5 | 3 | 3 | -17.4 | 100 | 100 |
|  |  | Mutant1 | 26 | 12156.11 ± 966.19 | 13920 | 1575.1 | 14 | 0 | -16.1 | 14.29 | 14.81 |
|  |  | Mutant2 | 37 | 12445.8±1069.31 | 15382 | 1590 | 10 | 6 | -10.9 | 62.5 | 45.28 |
|  |  | Mutant3 | 58 | 11976.6±669.02 | 13358 | 1476.3 | 10 | 4 | -1.1 | 18.75 | 16.98 |
|  |  | Mutant4 | 198 | 12554.71 ± 1277.78 | 14344 | 1693.6 | 7 | 7 | -10.4 | 14.29 | 25.93 |
|  | EXOSC8 | Wildtype |  | 13228.67±1288.05 | 15536 | 1747.6 | 3 | 2 | -18.1 | 100 | 100 |
|  |  | Mutant1 | 26 | 13380.67 ± 1044.25 | 15302 | 1889.9 | 9 | 1 | -19.3 | 31.15 | 45.28 |
|  |  | Mutant2 | 37 | 13263.33±1194.53 | 16304 | 1647.9 | 10 | 1 | -16.9 | 68.33 | 30.77 |
|  |  | Mutant3 | 58 | 12968.27±874.30 | 15194 | 1808.5 | 12 | 0 | -14 | 45 | 50 |
|  |  | Mutant4 | 198 | 13313.81 ± 1242.59 | 16010 | 1693.4 | 6 | 0 | -17.1 | 8.2 | 33.96 |
|  | SEPSECS | Wildtype |  | 14021±714.22 | 15318 | 1715.8 | 1 | 2 | -14.4 | 100 | 100 |
|  |  | Mutant1 | 26 | 14497 ± 1294.35 | 17544 | 2160.8 | 17 | 4 | -15.8 | 12.2 | 18.18 |
|  |  | Mutant2 | 37 | 15024.67±1444.97 | 17518 | 1932.6 | 8 | 3 | -24.1 | 30 | 5.56 |
|  |  | Mutant3 | 58 | 14201±1266.15 | 16714 | 2022.5 | 6 | 5 | -24.5 | 25 | 11.11 |
|  |  | Mutant4 | 198 | 14237.68 ± 1585.22 | 18278 | 2555.4 | 17 | 4 | -20.7 | 12.2 | 19.09 |
| NSP9 | GTF2F2 | Wildtype |  | 11369.5 ± 616.2 | 13622 | 1384.6 | 11 | 2 | -12.4 | 100 | 100 |
|  |  | Mutant 1 | 25 | 11494.52 ± 865.38 | 13518 | 1417.1 | 14 | 1 | -8.9 | 52.78 | 35.14 |
|  |  | Mutant 2 | 67 | 11833.73 ± 646.01 | 14242 | 1536.2 | 11 | 4 | -12.8 | 77.78 | 40.54 |
|  | MAT2B | Wildtype |  | 11698.74 ± 723.11 | 13262 | 1679.1 | 12 | 7 | -6.2 | 100 | 100 |
|  |  | Mutant 1 | 25 | 11838.1 ± 1005.91 | 14380 | 1407 | 9 | 1 | -6.7 | 14.06 | 71.43 |
|  |  | Mutant 2 | 67 | 12017.62 ± 637.43 | 13322 | 1711.4 | 17 | 0 | -4.3 | 0 | 83.33 |
| NSP10 | AP2M1 | Wildtype |  | 11237.55 ± 506.73 | 12774 | 1652.6 | 15 | 4 | -7.4 | 100 | 100 |
|  |  | Mutant1 | 32 | 12197.38 ± 721.38 | 13602 | 1876.4 | 15 | 0 | -9.1 | 4.26 | 0 |
|  | GFER | Wildtype |  | 12333.84 ± 1467.95 | 16656 | 1625.4 | 17 | 3 | -7.8 | 100 | 100 |
|  |  | Mutant1 | 32 | 12583.75 ± 1091.58 | 15156 | 1626 | 20 | 1 | -10.2 | 88.1 | 0 |
| NSP12 | PPIL3 | Wildtype |  | 12650.42 ± 825.02 | 14626 | 1526.7 | 9 | 9 | -8.8 | 100 | 100 |
|  |  | Mutant1 | 97 | 12942.69 ± 936.8 | 14846 | 1557.9 | 10 | 3 | -6.3 | 53.85 | 76.09 |
|  |  | Mutant2 | 323 | 12789.11 ± 934.38 | 16536 | 1754.8 | 7 | 5 | -11.3 | 17.95 | 63.04 |
| NSP13 | CEP250 | Wildtype |  | 12418.26 ± 909.84 | 15054 | 1632.5 | 14 | 2 | -10.1 | 100 | 100 |
|  |  | Mutant1 | 40 | 12447.88 ± 557.41 | 13920 | 1427.1 | 12 | 1 | -0.7 | 40 | 9.09 |
|  |  | Mutant2 | 237 | 12116.54 ± 792.2 | 14534 | 1778.1 | 19 | 4 | -9.8 | 66.67 | 7.27 |
|  |  | Mutant3 | 253 | 12212.83 ± 649.6 | 13050 | 1325.9 | 9 | 1 | -9 | 11.11 | 70.91 |
|  | FYCO1 | Wildtype |  | 12318.29 ± 906.91 | 15462 | 1729 | 14 | 3 | -5 | 100 | 100 |
|  |  | Mutant1 | 40 | 12565.16 ± 801.93 | 15260 | 1584.7 | 22 | 4 | -12.8 | 44.9 | 60.71 |
|  |  | Mutant2 | 237 | 12819.84 ± 1158.08 | 15984 | 1891.4 | 17 | 4 | -11.3 | 26.53 | 23.21 |
|  |  | Mutant3 | 253 | 12590.62 ± 989.93 | 15868 | 1653.8 | 11 | 1 | -10 | 63.27 | 41.07 |
| NSP14 | GLA | Wildtype |  | 17527.8 ± 1401.59 | 20826 | 1990.9 | 16 | 3 | -14.7 | 100 | 100 |
|  |  | Mutant1 | 119 | 17880.16 ± 1665.53 | 21500 | 2279.3 | 19 | 7 | -9.1 | 66.67 | 66.07 |
|  |  | Mutant2 | 177 | 15041.5 ± 625.87 | 16730 | 1788.6 | 18 | 4 | -8.3 | 56.86 | 33.93 |
|  |  | Mutant3 | 255 | 17269.58 ± 944.24 | 19026 | 1184.1 | 6 | 3 | -1.5 | 27.45 | 3.57 |
|  | IMPDH2 | Wildtype |  | 15757.05 ± 973.43 | 18234 | 2777.3 | 23 | 11 | -17.1 | 100 | 100 |
|  |  | Mutant1 | 119 | 16223.86 ± 1286.81 | 19782 | 2344.5 | 17 | 4 | -12.4 | 53.33 | 40 |
|  |  | Mutant2 | 177 | 15811.88 ± 884.65 | 17756 | 2596 | 17 | 5 | -11 | 70.67 | 48.42 |
|  |  | Mutant3 | 255 | 15813.28 ± 1086.36 | 19140 | 2326.7 | 22 | 2 | -8.9 | 8 | 26.32 |
|  | SIRT5 | Wildtype |  | 14332.55 ± 852.08 | 16476 | 1751.5 | 15 | 1 | -17 | 100 | 100 |
|  |  | Mutant1 | 119 | 14141.29 ± 787.96 | 15960 | 1887.5 | 12 | 2 | -14.4 | 4 | 69.49 |
|  |  | Mutant2 | 177 | 14774.63 ± 1117.4 | 16908 | 1733.5 | 8 | 5 | -19.7 | 46 | 52.54 |
|  |  | Mutant3 | 255 | 14405.2 ± 957.64 | 17150 | 1755.3 | 5 | 1 | -18.2 | 50 | 64.41 |
| NSP15 | ARF6 | Wildtype |  | 13112.88 ± 1274.99 | 16388 | 1662.1 | 5 | 3 | -7.8 | 100 | 100 |
|  |  | Mutant 1 | 23 | 12856.89 ± 756.55 | 14630 | 1563.6 | 9 | 2 | -6.3 | 15.69 | 42.62 |
|  |  | Mutant 2 | 109 | 12563.64 ± 946.32 | 15698 | 1535.8 | 12 | 6 | -0.3 | 13.73 | 37.7 |
|  | NUTF2 | Wildtype |  | 13629.78 ± 470.5 | 14458 | 1419.2 | 15 | 10 | -19.7 | 100 | 100 |
|  |  | Mutant 1 | 23 | 14101.29 ± 677.36 | 15486 | 1455.7 | 12 | 3 | -1.8 | 15.38 | 13.95 |
|  |  | Mutant 2 | 109 | 12444 ± 631.17 | 13834 | 1621.2 | 11 | 1 | -12.6 | 10.64 | 60 |
|  | RNF41 | Wildtype |  | 13229.29 ± 1286.85 | 16396 | 1878.9 | 15 | 3 | -4.4 | 100 | 100 |
|  |  | Mutant 1 | 23 | 13646 ± 1377.45 | 17674 | 1746.9 | 13 | 7 | -3.7 | 90.38 | 59.62 |
|  |  | Mutant 2 | 109 | 13409.84 ± 1215.4 | 16816 | 1869.7 | 13 | 5 | -11 | 82.69 | 61.54 |
